# Raising girls and boys in early China: Stable isotope data reveal sex differences in weaning and childhood diets during the eastern Zhou era

**DOI:** 10.1002/ajpa.24033

**Published:** 2020-03-06

**Authors:** Melanie J. Miller, Yu Dong, Kate Pechenkina, Wenquan Fan, Siân E. Halcrow

**Affiliations:** ^1^ Department of Anatomy University of Otago Dunedin New Zealand; ^2^ Archaeological Research Facility University of California Berkeley California USA; ^3^ Institute of Cultural Heritage Shandong University Qingdao China; ^4^ Department of Anthropology Queens College New York City New York USA; ^5^ Research Division of Shang and Zhou Dynasties, Henan Provincial Institute of Cultural Relics and Archaeology Zhengzhou China

**Keywords:** dentin, gender, incremental isotopic analysis, wheat, millet

## Abstract

**Objectives:**

Using stable isotope analysis of incremental dentin segments, we reconstruct breastfeeding, weaning, and childhood dietary patterns of Eastern Zhou period (771–221 BC) individuals from the Central Plains of China. Previous isotopic research on the Eastern Zhou demonstrated dietary difference between male and female diets in adulthood via bone collagen analysis. To understand the development of gendered dietary patterns we must examine the early life period. We aim to identify the timing of the weaning process, whether childhood diets were the same as adulthood diets, and if there were differences between the diets of boys and girls during childhood.

**Materials and Methods:**

We present incremental dentin and bone collagen δ^13^C and δ^15^N isotope data from 23 individuals from two Eastern Zhou archaeological sites (Xiyasi 西亚斯and Changxinyuan 畅馨苑).

**Results:**

Weaning was completed between ages 2.5 and 4 years. Females were weaned slightly earlier than males. Early childhood diets show significant incorporation of C_3_ foods, such as wheat and soybean, for almost all children, while later adulthood diets indicate greater incorporation of C_4_ foods (millets), particularly for males.

**Discussion:**

Childhood diets included greater amounts of C_3_ foods than expected, suggesting that grains such as wheat may have been adopted in these communities as foods for children. Nevertheless, dietary differentiation between females and males began in childhood, with boys eating more millets (C_4_ foods) than girls. The findings suggest that feeding children was a significant aspect of socialization and cultural gendering of individuals in ancient China.

## 1 INTRODUCTION

Growth, maintenance, and reproduction are three imperative functions of a living organism. Resource availability during infancy and childhood shapes the energy allocation toward each of these functions and determines the organism's survival into adulthood, adult health status, as well as the reproductive success of the individual (Gluckman, Hanson, & Beedle, 2007; Gluckman, Hanson, & Buklijas, 2010). The increasing impact of life‐history theory on bioarchaeological research has brought greater attention to studying children and childhood (Beauchesne & Agarwal, 2018; Halcrow & Tayles, 2011; Lewis, 2007; Perry, 2005; Temple, 2019). Studies of diet, health, and disease have been central to understanding childhood in the past, and can reveal critical effects that the care for children has in societies over time and space, and for human evolution at large (Fuller, Richards, & Mays, 2003; Goodman & Armelagos, 1989; Gowland, 2015; Humphrey, 2010; Katzenberg, Herring, & Saunders, 1996; Lewis, 2007; Moffat & Prowse, 2018; Schurr, 1998; Tsutaya, 2017). Dietary studies provide insights to anthropological questions related to how food practices are entangled in the social–political processes within any particular culture and can provide unique insights into the socialization of individuals through food consumption (Chang, 1977; Hastorf, 2017; Ohnuki‐Tierney, 1993; Sterckx, 2005; Twiss, 2007; Weismantel, 1988). Although most archaeological studies of dietary practices using methods such as stable isotope analysis have traditionally focused on adulthood diet (primarily a function of methodological progression and theoretical orientation over time), increasingly bioarchaeologists are recognizing the importance of the childhood period for revealing the biocultural position that food uniquely plays in human existence.

In recent decades anthropological isotopic specialists have increasingly turned to human teeth, which potentially provide resolution of human diets on the level of months to years (Burt, 2015; Eerkens, Berget, & Bartelink, 2011; Miller, Agarwal, & Langebaek, 2018; Reitsema & Vercellotti, 2012; Sealy, Armstrong, & Schrire, 1995; Turner, Kingston, & Armelagos, 2010; Wright & Schwarcz, 1999). Through incremental sampling of specific teeth, which form during discrete periods of development, bioarchaeologists can target particular periods of early life to understand maternal diet, breastfeeding, and weaning patterns in infancy, and dietary shifts during childhood, adolescence and early adulthood (Beaumont, Gledhill, Lee‐Thorp, & Montgomery, 2013; Burt & Garvie‐Lok, 2013; Dal Martello et al., 2018; Fernández‐Crespo, Czermak, Lee‐Thorp, & Schulting, 2018; Halcrow et al., 2018; King et al., 2018). Comparing dental isotopic results to bone samples from the same individual allows us to study dietary patterns over a lifetime and provides detailed individual histories of ancient peoples (Sealy et al., 1995). The results of these analyses can provide unique insights into social processes that are often obscured from other archaeological data, such as the relationships between food access and variables such as sex, gender, age, social status, and biological processes such as how diet and nutrition influence growth, development, and disease.

**Figure 1:**
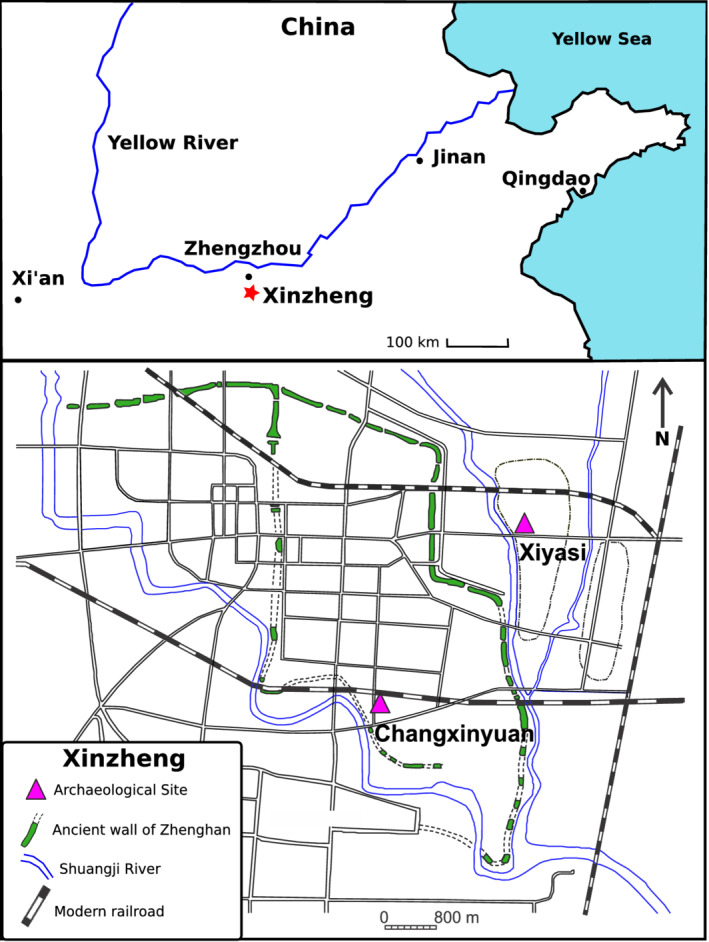
Map showing the location of the modern city of Xinzheng on China's Central Plains with the detailed location of the archaeological sites studied (Xiyasi and Changxinyuan in pink triangles) relative to the ancient Zhenghan city walls (in green segments) and Shuangji river (in blue)

This study examines the childhood diet of humans who survived into adulthood from two urban mortuary populations excavated from the Eastern Zhou (771–221 BC) city of Zhenghan (郑韩故城), located in the Central Plains region of China (Figure 1). Recent isotopic analysis of bone collagen from adult skeletons of Zhenghan documented significant differences in diets between adult females and males (Dong et al., 2017). Our research studies dietary patterns during early life through the incremental sampling of tooth dentin collagen using the stable isotopes of carbon and nitrogen. We targeted early‐developing permanent dentition, a first molar or canine tooth, to capture periods of dental development and therefore childhood diet, from birth up to ~14 years of age. We test whether these dietary differences between the sexes can be traced to the early phases of childhood and whether sex‐specific cultural practices pertaining to the timing of weaning, weaning foods, and childhood dietary patterns can be identified in early forming dental tissues.

### 1.1 Reconstructing individual dietary histories using stable isotope analysis

Breastfeeding and weaning are unique processes in humans because they reveal the deep entanglements of biology and culture (Cassidy & Tom, 2015; Maher, 1992; Moffat & Prowse, 2018; Stuart‐Macadam, 1995; Tomori, Palmquist, & Quinn, 2018). How babies are fed so that they can grow and develop healthily is critical to the continuity of a family, culture, and the survival of our species (Dettwyler, 1995, 2004; Humphrey, 2010). Breast milk is the primary dietary component for infants, providing rich nutrition and complex immunological support to the developing body (Andreas, Kampmann, & Mehring Le‐Doare, 2015; Hanson, 1998; Hanson et al., 2002; LaTuga, Stuebe, & Seed, 2014; Martin, 2018; Miller, 2018; Quinn, 2018). Over variable periods of time, babies are weaned so that they are not solely reliant on milk for their caloric and nutritional needs, with other foods introduced at different times during development, and finally cessation of breast milk consumption when the child is fully weaned. Furthermore, feeding children solid foods is biologically risky during early life; with their developing immune systems children are more prone to infection, some of which could be spread through poor food hygiene. The rapidly growing bodies of infants also need adequate caloric intake to ensure “normal” development of the body (such as reaching average height for a particular population). Variations in infant feeding practices (such as length of time breast milk is the exclusive food source) can have significant impacts on health outcomes (Dieterich, Felice, O'Sullivan, & Rasmussen, 2013; Miller, 2018; Moffat & Prowse, 2018; Victora et al., 2008, 2016). The timing and duration of the weaning process and the foods that are introduced to the child at different times, are heavily influenced by cultural beliefs, with potentially great differences between individuals, families, and cultural groups over time and space (Cassidy & Tom, 2015; Maher, 1992; Martin, 2018; Moffat & Prowse, 2018; Sobonya, 2018; Stuart‐Macadam, 1995; Veile & Kramer, 2018). Many of the practices related to feeding children are deeply tied to societal beliefs about how a body is properly nourished to grow and develop, and studying childhood dietary practices can provide significant insights into these aspects of culture. Therefore, studying dietary practices of children can indicate patterns where nutrition and health outcomes may be correlated, producing valuable information not only about the past societies we study, but serve as critical data with implications for modern populations.

Bioarchaeologists are able to contribute to our understanding of the patterns of human diet in ancient populations through methods including studies of skeletal chemistry, paleopathology, dental calculus composition, coprolite analysis, dental microwear, oral health, and more (Larsen, 2015). Bones and teeth record aspects of diet from the time periods they are formed and stable isotope analysis of these tissues can reveal dietary patterns from different periods of an individuals' life (DeNiro, 1985; Eerkens et al., 2011; Price, Schoeninger, & Armelagos, 1985; Sealy et al., 1995). Stable isotopes of carbon (δ^13^C) and nitrogen (δ^15^N) are now commonly used in archaeological studies because these elements provide important insights into human history and evolution, especially related to dietary patterns and paleoenvironmental reconstructions (Ambrose, 1993; DeNiro, 1985; Lee‐Thorp, 2008). Carbon and nitrogen stable isotope values relate to the foods people consumed during life, and are often preserved in skeletal tissues (DeNiro & Epstein, 1978; DeNiro & Schoeninger, 1983). Carbon provides information about the broad groups of plants that animals consumed during their life: modern C_3_ plants have δ^13^C values averaging around −23‰, while C_4_ plants have δ^13^C values averaging around −13‰ (DeNiro & Epstein, 1978; Ehleringer & Cerling, 2002; Farquhar, Ehleringer, & Hubick, 1989; Kohn, 2010; O'Leary, 1988). Nitrogen isotopic data relates to protein consumption and can indicate one's position within a local food web, with a 3–5‰ stepwise enrichment in nitrogen as one rises from the base of the food chain to top‐level predators (DeNiro & Schoeninger, 1983; Minagawa & Wada, 1984; Schoeninger & DeNiro, 1984). Nitrogen isotope values are also influenced by unique environmental conditions such as altitude and aridity, and nitrogenous inputs such as fertilizers (Ambrose, 1991; Bogaard, Heaton, Poulton, & Merbach, 2007; Szpak, 2014; Szpak, Millaire, White, & Longstaffe, 2012).

Bones and teeth are composed of two interwoven matrices, the organic collagen, and inorganic hydroxylapatite matrices, and each provides different isotopic data and reflects different aspects of diet. The organic collagen component has been found to preferentially incorporate dietary proteins, with amino acids routed directly from protein sources into the body's collagen matrix (Ambrose & Norr, 1993; Tieszen & Fagre, 1993). Dietary studies of hydroxylapatite (bone apatite, dental enamel) provide carbon isotope data (from carbonate incorporated into the structure) and this carbon has been found to be pulled from all carbon sources within the diet (so all major dietary components can contribute carbon to the inorganic fractions). Additionally, there are quantified offsets between the diet an organism consumes and the isotopic composition of bodily tissues, with collagen‐diet spacing of approximately +5‰ for δ^13^C (collagen δ^13^C value is +5‰ more positive than average diet) (Lee‐Thorp, Sealy, & van der Merwe, 1989; Sullivan & Krueger, 1981; Van Der Merwe & Vogel, 1978), and apatite‐diet spacing has been found to range from +9 to +12‰ for δ^13^C (Krueger & Sullivan, 1984; Lee‐Thorp et al., 1989). This study focuses on the organic collagen fraction of bone and tooth samples.

Babies exclusively consuming breast milk show a positive offset of +2–3‰ for δ^15^N and about +1‰ for δ^13^C compared with their mothers, as revealed in studies of fingernail samples from modern mother–baby pairs (Fogel, Tuross, & Owsely, 1989; Fuller, Fuller, Harris, & Hedges, 2006). Growing infants require dietary supplementation around the age of 6 months, as energetic requirements begin to outpace the nutritional content of breast milk (Kramer & Kakuma, 2002; Moffat & Prowse, 2018). As other foods are introduced and the baby is progressively weaned off breast milk the child's isotopic values will change, usually with a noted decline in δ^15^N, and at least a slight shift in δ^13^C (Eerkens et al., 2011; Fuller et al., 2003; Wright & Schwarcz, 1999), though other isotopic patterns related to a lack of breastfeeding and physiological stress have been noted in archaeological populations (Beaumont & Montgomery, 2016; King et al., 2018; Kwok, Garvie‐Lok, & Katzenberg, 2018). Previous bioarchaeological research has used patterned changes in δ^15^N (a drop of 2–4‰) and δ^13^C (decline by around 1‰) in tooth and bone samples to demarcate the completion of the weaning process and the shift to a diet without significant amounts of breast milk (Beaumont & Montgomery, 2016; Burt & Garvie‐Lok, 2013; Eerkens et al., 2011; Eerkens, de Voogt, Dupras, Francigny, & Greenwald, 2018). Therefore, we expect to see changes to the diet around 6 months of age with the introduction of new food sources, and likely expansion of dietary components as the child ages, with the underlying assumption that children eventually consume a diet that is very similar to the adults around them (Tsutaya, 2017).

Interpretations of estimated age when breast milk consumption is terminated are most successful in cases where childhood and adulthood diets were relatively homogeneous (i.e., where children are transitioned onto diets that appear very similar to adulthood diets), and where diets were usually isotopically constrained with very minimal mixing across C_3_, C_4_, terrestrial, and aquatic resources (Burt & Garvie‐Lok, 2013; Eerkens et al., 2018; Tsutaya, 2017). Identifying a specific age when weaning concludes can be difficult when individuals (in this case usually assumed as lactating mothers and their breastfeeding infant) eat across both C_3_ and C_4_ food groups (causing greater change in δ^13^C than the usual 1‰ expected decline) and where various protein sources may be introduced to children's diets (potentially confounding the drop in δ^15^N expected with the cessation of breast milk). Additionally, if children are weaned onto diets that are unique to the childhood period (and are different from the dietary values observed in adulthood for the same population), then isotopic profiles will not match the isotopic values of that same individual in later adult bone collagen samples, or the specified adult group dietary mean values (such as the average values of reproductive‐age adult females from the population), potentially confounding interpretation of an exact age where breast milk was no longer consumed. Finally, identifying smaller contributions by particular food groups will be isotopically masked by the more dominant food(s), such that if breast milk is retained in the diet but consumed in very small amounts (or consumed irregularly) it likely will not register in dentin chemistry. Therefore, we can at best make an estimate of a general time period where breast milk was significantly reduced and therefore other foods became more primary in the child's diet (Halcrow et al., 2018).

Due to differential renewal/turnover rates of bodily tissues, we can chemically analyze various tissues to document dietary inputs from particular periods of time during the life‐course. For example, teeth develop during very specific windows of childhood and record the chemical signature of the diet consumed during that time of growth (Beaumont et al., 2013; Burt & Garvie‐Lok, 2013), and then do not remodel after they are formed, creating a static tissue (however, localized chemical changes can occur due to disease or trauma). Additionally, since teeth develop at generally well‐defined chronological ages with deciduous teeth beginning in utero, and permanent third molars completing formation usually in late teens to early 20s, we can select particular teeth to target specific periods of growth and development to answer questions about early life dietary practices (Beaumont et al., 2013; Burt & Garvie‐Lok, 2013; Eerkens et al., 2011). Using teeth, we can address questions of maternal‐child interactions (breastfeeding and weaning) and child‐rearing related to food practices, and also look at dietary changes over different periods of youth. Human bone continues to remodel over the entire lifetime, and chemically reflects the average diet of at least the decade prior to death (Hedges, Clement, Thomas, & O'Connell, 2007). If an adult is sampled, their bone can be compared to one or more of their teeth, and potentially very detailed isotopic life histories can be produced, documenting dietary change or stability over the lifetime (Sealy et al., 1995; Turner et al., 2010). Additionally, biosocial experiences, such as sex, gender, age, and status can also be studied in relation to dietary patterns and can inform us of how these aspects of humanity were experienced in different cultures across time and space (Eerkens & Bartelink, 2013; Miller et al., 2018; Reitsema & Vercellotti, 2012; White, 2005).

Bioarchaeologists have generally used two strategies to study childhood dietary practices: analysis of bones from individuals who died during youth (usually sampling across different age groups from infants to adults to capture a cross‐section of dietary patterns); while others analyze teeth from humans of any age to capture discrete periods of early life and compare that to a bone sample to look at diachronic intra‐individual dietary patterns (see Beaumont et al., 2018 for a recent report on comparing these data sets). For example, Xia et al. (2018) examined rib and long bone samples from individuals from the Western Zhou Dynasty (1122–771 BC) site of Boyangcheng (薄阳城) from Anhui Province, China, for carbon and nitrogen isotope data to study dietary habits from different periods of life, including identifying breastfeeding and weaning practices. Testing a cross‐section of the mortuary population ranging from age 2 to 45+ years at time of death, the authors found that adults consumed diets with mixtures of C_3_ and C_4_ foods (individuals older than 14 years of age average δ^13^C –17.6‰ ±2.0‰; average δ^15^N 10.6‰ ±0.6‰) (Xia et al., 2018). They used the range of adult values as the comparative group for the young children, and found that by around ages 3 to 4 years their δ^15^N values dropped to values similar to the adults, and therefore the authors propose this is the age where weaning finished (Xia et al., 2018). They noted that children between 2 to 10 years of age also had diets slightly distinct from the adult population (lower in both δ^13^C and δ^15^N), suggesting that children ate more plant‐based diets.

The other method for studying childhood diet uses dentition to study discrete periods of time during early life, from either deciduous dentition or permanent dentition depending on the research question. For example, Yi et al. (2018) used both bone and tooth samples from a Late Neolithic population from the Gaoshan Ancient City (高山古城) in Sichuan Province, China. Their results found dietary variation over the lifetime of the individuals studied: the Neolithic Gaoshan adulthood diets ranged from δ^13^C –19.6 to −18.0‰, therefore dominated by C_3_ foods (rice), while their childhood diets ranged from δ^13^C –19.6 to −15.0‰, containing an unexpected signal indicating some consumption of C_4_ foods (millets) (Yi et al., 2018). Additionally, their incremental analysis of dentin showed that weaning occurred in that population between 2.5 and 4 years of age. These two studies show the potential for these different life‐course approaches to reveal detailed dietary patterns for individuals and populations (Xia et al., 2018; Yi et al., 2018).

Our study sampled permanent dentition from individuals who died as adults (defined here as completion of longitudinal growth as indicated by epiphyseal fusion of long bones) along with a bone sample from the same person. By studying individuals who died in adulthood, variables such as sex could be inferred (from the examination of sexual dimorphism of the pelvis and skull) and used as a factor to study how sex may relate to weaning patterns, childhood diet, and adulthood diet. We employ the incremental method of sampling human dentition, which divides a tooth into small segments from crown to root with associated age estimations for each section (Beaumont & Montgomery, 2015). This approach has demonstrated that dental incremental samples can provide dietary data over identifiable chronological periods on the order of months to years during specific times of childhood (Beaumont & Montgomery, 2016; King et al., 2018).

### 1.2 Human diet during the eastern Zhou period

The Zhou Dynasty comes to power in 1046 BC and maintains political control over a large territory in north‐central China for hundreds of years. The Zhou Dynasty is divided into Western Zhou (1046–771 BC) and Eastern Zhou (771–221 BC) periods. The Eastern Zhou is subsequently divided into two phases: the Spring and Autumn phase (771–476 BC) and the Warring States phase (475–221 BC). Eastern Zhou society was socially stratified with centralized power (king, nobles and regional rulers, commoners) but major states were increasingly factionalized and battled over time, culminating in the defeat of the Zhou dynasty and the unification of China by Qin Shihuang in 221 BC (Feng, 2013; von Falkenhausen, 2006). During the Zhou periods, urban centers increased in size and density over time, and people were engaged in various kinds of labor across subsistence work (such as crop cultivation and animal husbandry) and diverse craft specializations in pottery, jade, bronze, iron, textiles, and more (Feng, 2013; von Falkenhausen, 2006). Additionally, the Eastern Zhou period is a noteworthy time of Chinese intellectualism, with Confucius, Mencius, and other scholars living during this era, marking the beginning of their influential roles in Chinese culture for millennia (Feng, 2013; Hinsch, 2018; von Falkenhausen, 2006).

The Eastern Zhou archaeological sites of Xiyasi (西亚斯)and Changxinyuan (畅馨苑, previously written as 畅馨园, see Dong et al., 2017) are located within the Central Plains region of China, which encompasses the floodplain of the middle and lower reaches of the Yellow River. Two species of millet (common/broomcorn millet: *Panicum miliaceum*; foxtail millet: *Setaria italica*) were grown on the Yellow River floodplain, starting from about 10,000 years ago, with agricultural intensification of their cultivation in subsequent millennia (Cohen, 2011; Liu, Hunt, & Jones, 2009; Zhao, 2011). Around the same time period rice (*Oryza sativa*) was domesticated to the south of this area, in the Yangtze River valley region (Fuller et al., 2009; Jiang & Liu, 2006). Over time, rice agriculture expanded northward and millet agriculture moved south, so that populations living in a large swath of eastern‐central China potentially had access to both of these food groups by around 6000 BC (He, Lu, Zhang, Wang, & Huan, 2017; Wang, Lu, et al., 2018). Wheat (*Triticum* spp.) and barley (*Hordeum vulgare*) were introduced into the Central Plains of China around the late Neolithic and were increasingly incorporated into human diets over time (Chen, 2016; Deng et al. 2020; Guo & Jin, 2019; Liu et al., 2014; Zhou & Garvie‐Lok, 2015). Many studies have tracked the processes of domestication, intensification, and local incorporation of these various crops in different regions of China through archaeobotanical and stable isotope studies (some recent research: Liu et al., 2014; Guedes, Jin, & Bocinsky, 2015; Stevens et al., 2016; Dong et al., 2017; Dal Martello et al., 2018; Deng, Hung, Fan, Huang, & Lu, 2018; He, Lawson, Bell, & Hui, 2020; Song, Wang, & Fuller, 2019).

Most plants humans consume are C_3_ plants, including rice, soybeans, wheat, and barley, which have δ^13^C values around −23‰. Fewer domesticates, such as millets, are C_4_ plants, and have δ^13^C values around −13‰ (DeNiro & Epstein, 1978; Farquhar et al., 1989; Kohn, 2010; O'Leary, 1988). An, Dong et al. (2015) studied modern and archaeological millet samples from north‐central China and reported the modern millets had δ^13^C values ranging from −14.3 to −11.3‰ (with significant overlap between the two species), while archaeological millet samples had δ^13^C values ranging from −11.8 to −9.6‰ (again with significant overlap of the two species). The difference in carbon isotope values is primarily derived from temporal variation in atmospheric ^13^C, the Suess effect, with modern atmospheric δ^13^C about 1.5‰ more negative than preindustrialization due to the combustion of fossil fuels (Keeling, 1979; Marino & McElroy, 1991). Recent research on archaeological millet samples from northern China dated to the Late Neolithic by Wang, Fuller, et al. (2018) provides additional carbon and nitrogen isotope data for these species. Wang et al. (2018) also found that the δ^13^C values for foxtail and common millet overlapped, with foxtail having the larger range (−11.5 to −8.4‰) and encompassing the common millet range (−10.3 to −9.2‰). They also noted that the nitrogen isotope values overlapped, with common millet spanning δ^15^N 3.3 to 6.6‰ and foxtail millet ranging from δ^15^N 3.9 to 6.9‰ (Wang, et al., 2018). The archaeological millet nitrogen isotope values were higher than expected, and the authors suggested these crops may have been manured with animal dung (Wang, Fuller, et al., 2018).

The isotopic difference between these C_3_ and C_4_ plant groups allow archaeologists to track the incorporation of these plants into human diets. The Central Plains of China were traditionally dominated by millet agriculture but it is during the Eastern Zhou period when wheat may have become increasingly part of human dietary practices, paving the way for the future dominance of these food products (Dong et al., 2017; Zhou & Garvie‐Lok, 2015). Archaeobotanical samples from Xingyang Guanzhuang (荥阳官庄, dated to Western Zhou) and Dengfeng Wangchenggang (登封王城岗, dated from Late Neolithic to Bronze Age, with an Eastern Zhou component) report large proportions of foxtail millet (Lan & Chen, 2014; Zhao & Fang, 2007). In addition to this agricultural staple, plants such as wheat, soybean, broomcorn millet, and rice have also been recovered in archaeobotanical samples from Zhou sites in the Central Plains (Lan & Chen, 2014; Zhao & Fang, 2007). Current evidence suggests the importance of rice dropped considerably in the Central Plains region from the late Neolithic to Bronze Age while wheat gained importance (Liu, Song, & Gong, 2017; Wu, Zhang, & Jin, 2014; Yang, Yuan, & Zhang, 2017; Zhao & Fang, 2007). Wheat has become the second most commonly found cereal during Zhou Dynasty periods, with soybean being the third (Lan & Chen, 2014; Zhao & Fang, 2007). Barley is rarely found in the Central Plains region from the late Neolithic to Bronze Age. Unfortunately, no archaeobotanical studies were conducted for the two sites examined here (Xiyasi and Changxinyuan), therefore we rely on results from other Zhou period sites to help contextualize our isotopic findings.

Zooarchaeological studies of Central Plains archaeological sites have indicated the importance of various domesticated and wild animals, including terrestrial and aquatic species. The faunal assemblages of Eastern Zhou period sites are dominated by pig bones, with some contributions from dog, cattle, horse, sheep, and smaller amounts of fish, deer, turtle, bird, and other animals (Cao & Wang, 2016; Luo, Yang, & Yuan, 2005). A small number (*n* = 22) of faunal bones have previously been analyzed for stable carbon and nitrogen isotope data (Table S1) from two urban Eastern Zhou sites (Changxinyuan and Tianli) in ancient Zhenghan city (Dong et al., 2017; Dong personal comm.). The species analyzed include pig, dog, cattle, and sheep and show a large range of isotope values across the samples: δ^13^C ranged from −18.6 to −6.8‰; δ^15^N ranged from 2.4 to 10.7‰. This range of values indicate diets spanned from exclusively C_3_ to exclusively C_4_ plants and varying combinations between. Pigs are thought to have been a primary protein source and the seven pig bones analyzed to date have an average δ^13^C of −11.7‰ (*SD* = 2.7‰) and average δ^15^N of 6.2‰ (*SD* = 1.0‰), suggesting most pigs consumed a mixed diet of C_3_ and C_4_ plants, and that they may have been foddered on significant amounts of millet plants.

Dong et al. (2017) compared stable isotope values from human bone samples between Neolithic and Eastern Zhou period archaeological skeletal populations from the Central Plains region. They found that during the Neolithic, male and female isotopic values overlapped, suggesting large proportions of C_4_ millets in human diets, and generally similar access to animal proteins for both males and females. Conversely, based on data from urban archaeological sites from the Eastern Zhou period, male and female diets were statistically significantly different, with males consuming greater proportions of C_4_ plants and animal products than females (Dong et al., 2017). These dietary differences between males and females were coupled with a considerable increase of the adult body height dimorphism during the Eastern Zhou and an overall inferior funerary treatment of women, signaling their diminished social status (Dong et al., 2017). Drawing on their results, data from other Eastern Zhou sites, and historic records, the authors suggested that indigenous millets were perceived as more desirable and higher value staple foods during the Eastern Zhou, compared to more recently introduced wheat and beans (Dong et al., 2017).

Expanding on these findings of dietary differences between females and males, we set to test whether male–female dietary inequality can be traced to the earliest phases of childhood. Male–female dietary differences may manifest as different timing of weaning as well as differences in weaning and childhood diets. We sampled 23 individuals from the Eastern Zhou period urban mortuary populations of Xiyasi (西亚斯) and Changxinyuan (畅馨苑), for a single, early developing, permanent tooth. We hypothesized that: (a) children were weaned between 2 and 4 years of age, showing a similar pattern to other archaeological populations that have been studied in China (Xia et al., 2018; Yi et al., 2018); (b) dietary differences between males and females began in childhood (i.e., patterned differences in δ^13^C and δ^15^N between the males and females from each site), with males consuming more C_4_ foods and females consuming more C_3_ foods.

## 2 MATERIALS AND METHODS

### 2.1 The archaeological context

The modern Chinese city of Xinzheng, 新郑, is built over the ancient city of Zhenghan, which was a capital of Zheng 郑国 and then Han 韩国 kingdoms during the Easter Zhou period (Henan Provincial Institute of Cultural Relics and Archaeology, 2012). Zhenghan city has extensive wall fortifications around the city, and many archaeological sites have been found both within and outside the city walls. Our samples come from two archaeological sites from the ancient Zhenghan city: Xiyasi and Changxinyuan. Burials at both sites ranged from simple earthen pits to elaborate double chamber tombs with diverse grave goods. Unfortunately, both sites were looted in the past, but burials with grave goods present included offerings of cowry and other shells, Eastern Zhou pottery, bone (pig, deer, sheep/goat), and jade and bronze objects (Henan Provincial Institute of Cultural Relics and Archaeology, 2012).

The site of Xiyasi (Figure 1) is located outside of the eastern city wall of ancient Zhenghan city (Henan Provincial Institute of Cultural Relics and Archaeology, 2012). It was excavated between 2006 and 2008 during the construction of part of the Xiyasi University campus, and 320 ancient burials were uncovered. The burials included male and female adults of all ages, with a diversity of mortuary treatments ranging from complex burials with multiple offering chambers, double coffins, and tiled rooms to simple burials (Table 2). Very few juveniles were recovered from Xiyasi. Grave goods, when present, included elaborate pottery and bronze vessels, metal and jade objects, beads, and animal bones. Burials were assigned to a time period based on associated artifacts such as pottery, with a few burials being directly dated. The Xiyasi burials span both the Spring and Autumn and Warring States phases of the Eastern Zhou period, but most of the burials are dated to the Warring States phase. Five Xiyasi skeletons were directly radiocarbon dated at Peking University AMS lab (Peking University No. 2017105), producing a range of radiocarbon dates spanning from 771 to 43 cal BC.

Changxinyuan was excavated between 2009 and 2012 and is found within the city walls of ancient Zhenghan city (Figure 1). Excavations at Changxinyuan uncovered 82 human burials (males and female adults of all ages and few juveniles) and ranged from simple to elaborate mortuary treatment. Burial goods included characteristic Eastern Zhou pottery and bronze vessels, metal and jade objects, beads, and animal bones. Burials from Changxinyuan were dated from associated artifacts to both the Spring and Autumn and Warring States phases of Eastern Zhou. Two skeletons were directly AMS radiocarbon dated and span from 750 to 206 cal BC (Peking University No. 2017105).

### 2.2 Materials

Tooth samples were selected based on multiple criteria to try to sample across the mortuary populations: (a) preference for individuals who had been included in the previous bone collagen isotopic study by Dong et al., 2017; (b) preference for individuals who had a preserved first molar or canine; (c) preference to include a similar number of female and male individuals; (d) preference to include a range of tomb types (Tables 1 and 2). Permanent first molars (M1) were preferentially selected because of their developmental timeline, beginning to form within the first months after birth and finishing around age 9–10 years, and permanent canines were the second choice and begin to form around age 6 to 9 months and are complete between 13 and14 years (AlQahtani, Hector, & Liversidge, 2010; Hillson, 2014; Ubelaker, 1989). All tooth samples were molded and photographed prior to sample preparation. Fifteen adult individuals from Xiyasi had a tooth for analysis (*n* = 7 females, *n* = 8 males) and eight adult individuals from Changxinyuan had a tooth for analysis (*n* = 4 females, *n* = 4 males). Additionally, there were four Changxinyuan individuals without existing bone collagen data so we included a bone sample from each for isotopic analysis (Table 2). Age and sex were assessed using standard osteometric methods examining the pelvis and skull (Brooks & Suchey, 1990; Buikstra & Ubelaker, 1994).

**Table 1:** 

Site	Sample ID	Sex	Estimated age at death (young = 18–29; middle = 30–49; older = 50+)	Tooth sampled	Numer of serial sections	Sample notes
Xiyasi	M016	Male	Middle adult	M1	11	
Xiyasi	M018	Female	Middle adult	M1	10	
Xiyasi	M040	Male	Middle adult	C	12	Tooth worn; combined slices 11&12
Xiyasi	M051	Female	Middle adult	M1	14	Combined slices 11&12 and 13&14
Xiyasi	M068	Male	Younger adult	M1	11	
Xiyasi	M088	Female	Middle adult	M1	13	Combined slices 12&13
Xiyasi	M094	Male	Younger adult	M1	14	Combined slices 13&14
Xiyasi	M121	Female	Younger adult	C	15	Combined slices 12&13 and 14&15
Xiyasi	M139	Male	Middle adult	C	15	Combined slices 12&13 and 14&15
Xiyasi	M157	Male	Older adult	C	13	Tooth worn; combined slices 12&13
Xiyasi	M162	Female	Older adult	M1	12	Combined slices 11&12
Xiyasi	M205	Male	Older adult	M1	10	Combined slices 9&10
Xiyasi	M237	Female	Middle adult	C	16	Tooth worn; combined slices 13&14 and 15&16
Xiyasi	M314	Male	Middle adult	C	14	Tooth worn; combined slices 11&12 and 13&14
Xiyasi	M350	Female	Older adult	M1	11	Combined slices 10&11
Changxinyuan	M026	Male	Older adult	M1	10	
Changxinyuan	M043	Female	Middle‐older adult	C	11	Tooth worn
Changxinyuan	M045	Female	Middle‐older adult	M1	11	
Changxinyuan	M048	Female	adult	M1	11	
Changxinyuan	M049	Male	Middle‐older adult	M1	10	Tooth worn
Changxinyuan	M065	Female	Middle‐older adult	C	14	Tooth worn; combined slices 13&14
Changxinyuan	M067	Male	Middle adult	C	14	Combined slices 11&12 and 13&14
Changxinyuan	M074	Male	Older adult	M1	11	

**Table 2:** 

Site	Sample ID	Sex	Estimated age at death (young = 18–29; middle = 30–49; older = 50+)	Grave goods	Coffin (tomb type)	Bone	Tooth	Estimated weaning age (in years)	Average δ^13^C dentin (post 5 years old; VPDB)	Average δ^15^N dentin (post 5 years old; AIR)	δ^13^C bone collagen (VPDB)	δ^15^N bone collagen (AIR)
Xiyasi	M018	Female	Middle	3	Outer	Fibula	M1	3.1	−10.2	8.5	−9.8	10.1
Xiyasi	M051	Female	Middle	0	Inner	Fibula	M1	2.3	−16.6	7.1	−15.1	7
Xiyasi	M088	Female	Middle	1	Inner	Humerus	M1	3.3	−17.7	8.4	−14.6	7.7
Xiyasi	M121	Female	Younger	1	Inner	Fibula	C	4.3	−17.5	7	−15.8	7.3
Xiyasi	M162	Female	Older	1	Inner	Fibula	M1	3.3	−14.3	7.9	−13.5	8.4
Xiyasi	M237	Female	Middle	6	Inner	Fibula	C	3.5	−14.5	6.5	−13.5	6.6
Xiyasi	M350	Female	Older	0	None	Fibula	M1	3.4	−12.9	6.9	−11.4	8.2
Xiyasi	M016	Male	Middle	2	Inner	Fibula	M1	3.8	−10.6	8.2	−8.9	10
Xiyasi	M040	Male	Middle	0	Inner	Fibula	C	3.6	−14.3	7.5	−14.4	7.6
Xiyasi	M068	Male	Younger	0	Inner	Fibula	M1	3.8	−14.2	9.1	−12.3	7.7
Xiyasi	M094	Male	Younger	1	Double	Fibula	M1	4.3	−13.5	9.2	−10.2	9.2
Xiyasi	M139	Male	Middle	0	Inner	Fibula	C	4.3	−16.1	7.3	−13.7	7.5
Xiyasi	M157	Male	Older	0	Double	Fibula	C	4.1	−13.9	6.1	−10	8.4
Xiyasi	M205	Male	Older	2	Outer	Fibula	M1	4	−12.3	8.5	−9.8	8.6
Xiyasi	M314	Male	Middle	0	Inner	Ulna	C	5.2	−13.9	6.6	−11.4	6.7
Changxinyuan	M065	Female	Middle to older	0	Inner	Fibula	C	3.9	−13.6	6.6	−11.4	7.9
Changxinyuan	M045	Female	Middle to older	2	Inner	Fibula	M1	2.9	−12.7	7.3	−10.3	7.5
Changxinyuan	M048	Female	Adult	0	Inner	Radius	M1	2.1	−15.5	5.6	−11.4	6.5
Changxinyuan	M043	Female	Middle to older	2	Inner	Bone	C	4.6	−14.8	5.6	−12.3	6.1
Changxinyuan	M026	Male	Older	3	Inner	Bone	M1	3.8	−9.5	7.2	−10.9	6.7
Changxinyuan	M049	Male	Middle to older	0	Inner	Bone	M1	4.2	−8	9.2	−9.6	7.5
Changxinyuan	M074	Male	Older	1	Inner	Fibula	M1	3.4	−11.1	7.3	−9.6	7.7
Changxinyuan	M067	Male	Middle	3	Inner	Bone	C	2.6	−10.5	7.3	−9.7	6.9

The two tooth types included in our analysis were first molars (*n* = 14) and canines (*n* = 9), which were divided into 1 mm increments after demineralization for isotopic analysis (Beaumont & Montgomery, 2015). Dentin is not deposited in perfectly horizontal layers, meaning that our slices of dentin will capture overlapping periods of dentin deposition (Beaumont et al., 2013; Hillson, 2014). Therefore, the isotopic results from a single incremental slice of dentin represent an averaged period of growth and development rather than a single point in time. We utilized the method developed by Beaumont and Montgomery (2015) to associate each sampled increment with a developmental age estimate. This method applies a simple relationship between documented tooth developmental stages with the timing of tooth growth and the number of samples taken by the researcher (AlQahtani et al., 2010; Beaumont et al., 2013; Beaumont & Montgomery, 2015). This calculation produces an expected median age of each serially sampled dentin area for each tooth, which is a standardized way of estimating age across different samples. The 23 teeth we sampled produced 283 incremental dentin sections, some of which were combined to reach minimum sample size for the mass spectrometer (Table 1) and others (*n* = 24 samples) did not yield sufficient samples for analysis, meaning there are occasional time gaps in the longitudinal tooth data (Table S2).

### 2.3 Methods

Dental samples were prepared for stable isotope analysis following published protocols (Beaumont & Montgomery, 2015; King et al., 2018). Enamel was removed using a dental drill and preserved for future analysis, tooth surfaces were cleaned to remove any adhering dirt, cementum, and secondary and/or tertiary dentin if present. Teeth were placed into glass beakers with 0.5 M HCl for demineralization in 4–6°C and the HCl was changed every 24–48 hr as needed. Once demineralized to a flexible texture, teeth were rinsed to neutral and then sectioned by hand into ~1 mm sections starting at the occlusal surface and working towards the root apex using a clean scalpel and a ruler. The sectioned samples were further demineralized in 0.5 HCl, then, gelatinized by heating in a pH 3 HCl solution at 75°C for 24 hr. This dentin preparation did not use NaOH treatment because: (a) samples were small and there was the concern of sample loss with additional chemical treatment and (b) the knowledge that tooth samples are usually preserved better than bone, and all but four individuals had previously analyzed bone samples that indicated well‐preserved collagen. The use of %C, %N, and C:N ratios was used to ensure all dentin samples were of good quality collagen (see below). Finally, the samples were freeze‐dried in preparation for isotopic analysis.

Most bone collagen data was previously reported in Dong et al. (2017) but a few bone samples (*n* = 4) were prepared for collagen following the same method (Ambrose, 1990; Dong et al., 2017). Long bone samples were preferentially selected (from broken elements) and cortical surfaces were cleaned using a dental tool. The bone was crushed, sieved and the 0.25 mm to 1 mm fraction was used for collagen preparation. Samples were placed in glass beakers with 0.2 M HCl and left at room temperature to demineralize, HCl was replaced as needed until demineralization was complete. Samples were rinsed with purified water and then treated with 0.125 M NaOH for 20 hr, then rinsed with purified water. Finally, 0.001 M HCl was added to each sample and then placed in a 75°C oven for 24 hr to gelatinize the collagen. Samples were then frozen and freeze‐dried.

Samples were analyzed at the Stable Isotope Laboratory of Shenzhen Huake Precision Testing Inc. in China. Samples were analyzed on a Flash 2000 Elemental Analyzer coupled to a Delta V Advantage Isotope Ratio Mass Spectrometer. The lab reports internal precision for δ^13^C to ±0.1‰ and for δ^15^N to ±0.2‰. Internal lab standards of collagen and urea were analyzed with the samples, and an internal quality control standard was also included (NIST SRM 1547 peach leaves). All archaeological collagen samples (Table S2) were assessed for quality using %C, %N, and atomic C:N ratios, and all samples had C:N values between 2.9 to 3.4, within the accepted range for biogenic preservation of collagen (DeNiro, 1985; van Klinken, 1999).

## 3 RESULTS

**Figure 2:**
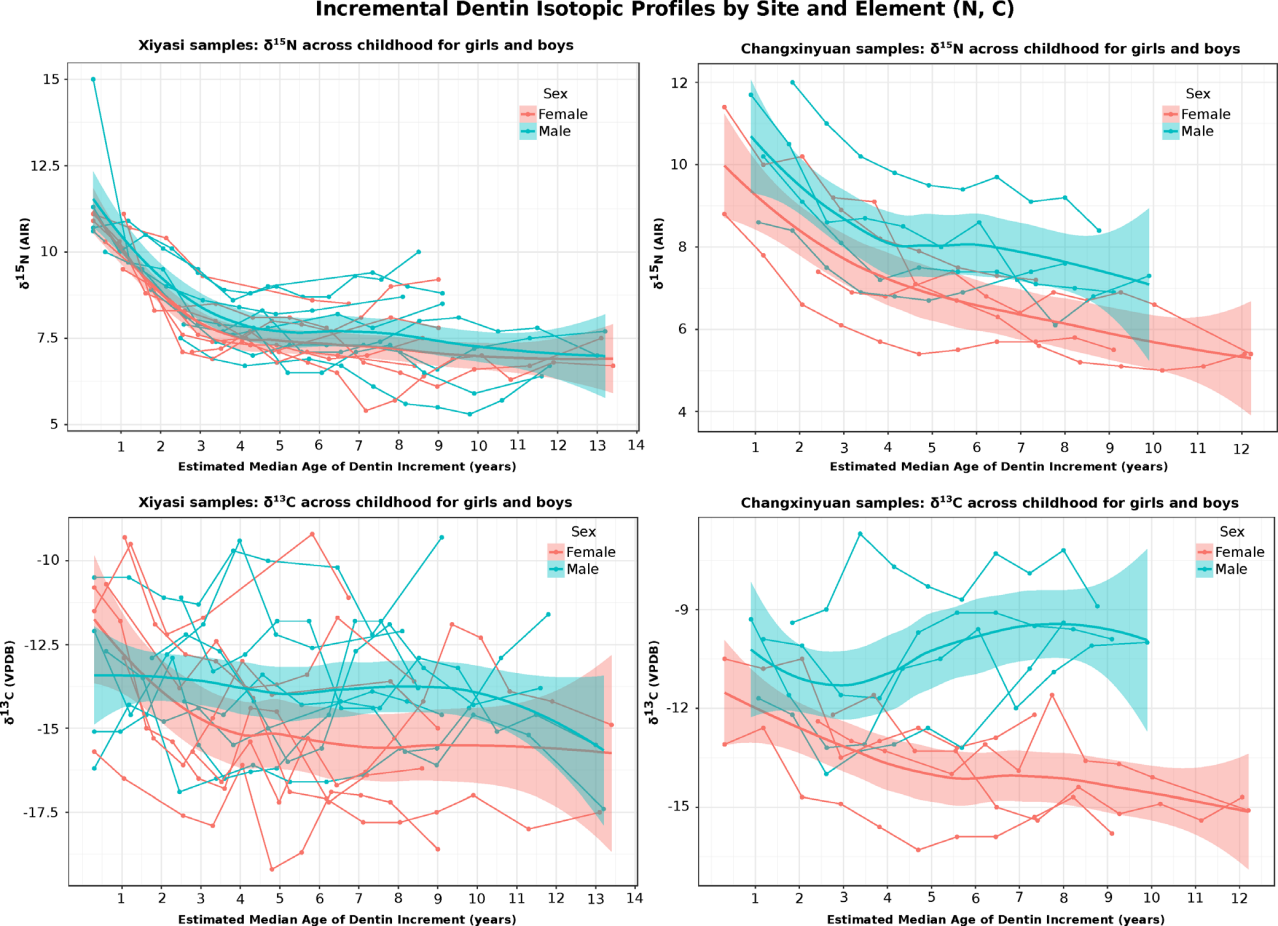
Stable isotope profiles from serial incremental dentin samples separated by site and element, with sex indicated by color (females in red, males in blue). The x‐axis is the estimated median age of each dentin sample in chronological years and the y‐axis is the stable isotope value for each sample (note some time periods are missing due to insufficient collagen yield or tooth wear). Loess curves (using the R “smooth” function) are plotted for each element at each site with the variable of sex to show patterns between diets of boys and girls during childhood

**Figure 3:**
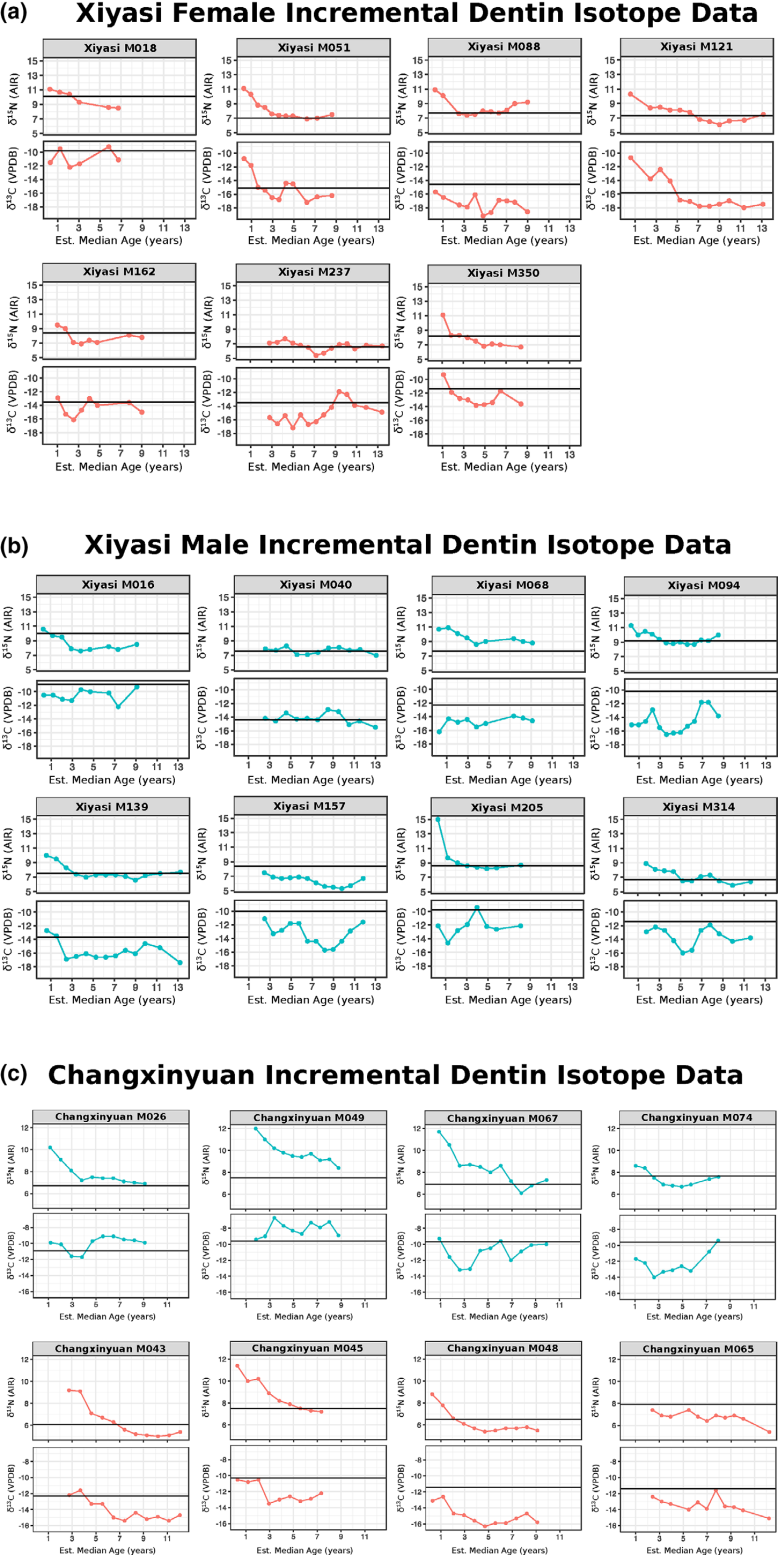
(a–c) Stable isotope profiles from serial incremental dentin samples separated by site and element, with sex indicated by color (females in red, males in blue). (a) shows Xiyasi females, (b) shows Xiyasi males, and (c) shows Changxinyuan males and females. The x‐axis is the estimated median age of each dentin sample in chronological years and the y‐axis is the stable isotope value for each sample (note some time periods are missing due to insufficient collagen yield or tooth wear). The solid black line is the same individual's adult bone collagen isotope value to allow comparison between childhood to adulthood for each individual (data from Dong et al., 2017 and this study)

Summary isotopic results are shown in Table 2, Figures 2 and 3, and complete dentin isotopic results are presented in Table S2. Overall, the dentin collagen δ^13^C values ranged from −19.2 to −6.7‰, and δ^15^N values ranged from 5.0 to 15.0‰. Within the Xiyasi samples the dentin δ^13^C values ranged from −19.2 to −9.2‰ and the δ^15^N from 5.3 to 15.0‰, while Changxinyuan individuals' dentin δ^13^C ranged from −16.3 to −6.7‰ and δ^15^N 5.0 to 12.0‰.

The corresponding bone collagen dietary data (most data previously reported in Dong et al., 2017) for these same individuals is reported in Table 2, with Xiyasi individuals' δ^13^C ranging from −15.1 to −8.9‰ (average δ^13^C = −12.3‰) and δ^15^N ranging from 6.6 to 10.1‰ (average δ^15^N = 8.1‰), and Changxinyuan samples' δ^13^C ranging from −12.3 to −9.6‰ (average δ^13^C = −10.7‰) and δ^15^N ranging from 6.1 to 7.9‰ (average δ^15^N = 7.1‰).

## 4 DISCUSSION

### 4.1 Weaning patterns

The dentin isotopic profiles of Xiyasi and Changxinyuan individuals demonstrate the difficulty of pinpointing a specific age of weaning in populations with varied food sources and a high degree of dietary diversity between individuals, and within the same individual over the lifetime. Weaning is a process rather than a singular moment in time. During the months to years that a child is weaned off breast milk, various foods are introduced into the infant's diet and eventually breast milk is completely removed as a dietary source. Developing infants have significant energy demands and supplementary foods must be introduced in order to provide sufficient caloric intake (WHO recommends at around 6 months of age).

All the isotopic profiles show elevated δ^15^N values for the first dentin increment and a subsequent decline in δ^15^N in the earliest forming increments over the first few years of life indicating the ubiquity of breastfeeding in early life for Eastern Zhou children (exceptions are a few individuals missing their earliest incremental periods due to occlusal tooth wear Xiyasi: M040, M157, M237, M314; Changxinyuan: M043, M049, M065). Most individuals also show a significant change in δ^13^C over their first few years of life, with changes between 2 and 6‰ noted across the individuals studied. This variability in δ^13^C during early life indicates that carbon sources in these very young childrens' diets were shifting, either because their mother's diet was changing (therefore altering the δ^13^C of the breast milk), or through the introduction of complementary weaning foods from different C_3_/C_4_ sources, or a combination of both.

Nine individuals (*n* = 7 Xiyasi and *n* = 2 Changxinyuan) have dentin isotopic profiles that follow the “typical” weaning curves that other studies have identified, with a 2–4‰ drop in δ^15^N which then generally flattens out, and a concurrent ~1‰ drop in δ^13^C (Beaumont & Montgomery, 2016; Burt, 2015; Eerkens et al., 2018; Jay, 2009; Jay, Fuller, Richards, Knüsel, & King, 2008). These individuals (from Xiyasi: M139, M088, M157, M162, M094, M016, M068; from Changxinyuan: M026, M067), show a range of ages where these isotopic changes occur, from about 2.5 years to 4 years of age, with the average age estimated at 3.7 years (across all nine individuals with this patterned profile). The other 14 individuals studied (*n* = 8 Xiyasi, *n* = 6 Changxinyuan) do not have isotopic profiles that neatly fit this “typical” weaning model, instead many show a protracted decline in δ^15^N over many years and significant fluctuations in δ^13^C, clouding the ability to make a very clear identification of the cessation of breast milk in the diet. For these individuals we made an estimation of the age of weaning based on patterns of δ^15^N declining in the early‐forming increments and concurrent changes in the δ^13^C in the isotopic profiles for these 14 individuals. We found that those estimated weaning ages ranged from about 2 to 5 years old, with an average estimated age of 3.6 years (which closely matches the average age for those nine individuals that had more “typical” weaning curves, see Figure S1). We, therefore, suggest that for the Eastern Zhou populations of Xiyasi and Changxinyuan, children stopped consuming breast milk between 2.5 and 4 years old.

This variation in weaning age within the Xiyasi and Changxinyuan samples suggest a range of breastfeeding and weaning practices for women and their babies. This age range agrees with the estimated weaning ages reported by Yi et al. (2018) from their dentin incremental isotopic analysis of five individuals from the late Neolithic Gaoshan site (southwestern China), and the weaning ages calculated by Xia et al. (2018) who studied bone collagen samples from Western Zhou period juveniles from Boyangcheng site in Anhui Province.

Comparing the weaning ages for the Xiyasi and Changxinyuan sites, we found the average estimated age of weaning completion at Xiyasi was 3.7 years while it was 3.4 years at Changxinyuan. This small difference in timing may be related to different practices within these groups, the small number of individuals understudy, and/or error associated with age estimation from dentin increments. Within each site we compared the estimated weaning age for females and males and found that there was very little difference between the sexes at Changxinyuan (estimated 3.4 years for Cxy females and 3.5 years for Cxy males), but that there is a pattern of difference between age of weaning for Xiyasi females (average age 3.3 years) and Xiyasi males (average age 4 years). On Figure 2 we plotted a loess curve for each sex (using the smooth function in R studio) and we can see that the nitrogen curves have an inflection point around that estimated average weaning age for each site and sex, and we also note that the curves for the females are lower than the males (see discussion section 4.2.1 below). It appears that Xiyasi mothers weaned their daughters off of breast milk earlier than they did their sons, possibly an indication of differential parental investment/feeding strategies based on the child' sex. Further studies with larger sample sizes may illuminate if these differences in breastfeeding duration between boys and girls are statistically significant, if this pattern appears in other Eastern Zhou communities, and if these practices are a shared cultural norm for ancient Chinese societies.

Interestingly, two historical sources provide further evidence to support the general weaning patterns observed in these Chinese archaeological isotopic studies to date. According to Analects of Confucius, which were initially recorded during the Warring States period (475–221 BC), but assumed their final form during the mid‐Han Dynasty (206 BC–AD 220), Confucius taught that deceased parents must be mourned for a 3‐year period, as this is the same length of time that parents care intensely for their children after birth: 宰我出，子曰:“予之不仁也!子生三年，然后免于父母之怀，夫三年之丧，天下之通丧也。予也有三年之爱于其父母乎?”(Chen, F. [Confucius], 2017, p. 215). The English translation reads: “Zai Wo then went out, and the Master said, “A man without human‐heartedness! Only when a child is three years old does he leave his parents' arm. The three years' mourning is the universal mourning under heaven. And Yu, did he not enjoy his parents' affection for three years?” This reference in Analects suggests that within Bronze Age Chinese society the age of three was an important social age where parent–child relationships and dependency may have changed (Chen, F. [Confucius], 2017), and perhaps concurrent transformations in mother‐dependent feeding also occurred during this period of time.

Xia et al. (2018) highlight the *Qianjinfang 千金方* text by Sun Simiao 孙思邈 (Sun AD?–AD 682), an influential physician of Sui and Tang Dynasties, and which is the earliest known document that discusses breastfeeding, weaning, and childhood feeding. They note that the *Qianjinfang* indicates that infants should be exclusively breastfed for 6 months, followed by the introduction of complementary foods (Sun, 1993 cited in Xia et al., 2018).

The isotopic data reported in this study suggests that urban Eastern Zhou families were generally following these practices, with all children breastfed as young infants and then breast milk consumption declined over the first few years of life with the completion of weaning by age 4, plus inclusion of diverse complementary foods over that period as well.

### 4.2 Dietary patterns over the life course

The isotopic profiles indicate significant variation in diet across childhood (Figure 3). This is not entirely surprising given the diversity of adulthood diets captured in the same individuals' bone collagen data (and the larger sampling of each population by Dong et al., 2017). The major food groups of millet, wheat, soybean, pig, cattle/ox, dog, horse, sheep, and more, transverse large ranges of δ^13^C and δ^15^N, and various combinations of these foods can produce the same isotopic value in skeletal tissues (this is the issue of equifinality, where different isotopic combinations can provide the same result, see King et al., 2018; Quinn, 2019). Therefore, interpreting changes in δ^15^N and δ^13^C becomes more challenging as we see the dietary diversity observed in Eastern Zhou adults is also reflected in their childhood diets.

#### 4.2.1 Nitrogen isotopes over the lifetime

For most of the individuals studied, after weaning concludes the δ^15^N dentin values flatten and remain relatively stable (almost all individuals show this pattern by age 5, therefore we use age 5 as the starting point for where children's diets no longer include breast milk). In most individuals' isotopic profiles (Figure 3), we see that the δ^15^N values from the later‐forming dentin samples are very close to, if not overlapping with, the δ^15^N value of the bone collagen sample representing their adulthood diet (average diet for the decade prior to death). This suggests relative stability in dietary protein sources after age ~5 years old through adulthood until death, though there may have been fluctuations during years that our samples do not account for. A few of the individuals from the Changxinyuan cemetery (Cxy M067, Cxy M049, Cxy M045), show more protracted declines in δ^15^N across their dentin profile, continuing past the estimated age of cessation of breastfeeding. This suggests their dietary protein intake was almost continuously changing across their childhood. However, even these individuals have their final dentin δ^15^N values approaching their bone collagen δ^15^N values, potentially suggesting relative stability in dietary protein from that point in later childhood onward in their lifetime.

We calculated the average of the dentin isotope values for each individual after age 5 (Tables 2 and S2) and then grouped the females and males and calculated the isotopic average for each sex during childhood. We found that Xiyasi females average δ^15^N post‐age 5 years was 7.5‰, and the Xiyasi males average δ^15^N was slightly higher at 7.8‰ (see loess curves on Figure 2, female curve is slightly lower than males over the entire childhood period covered by these samples). These data indicate protein consumption was relatively similar for both sexes from childhood through adulthood (i.e., no significant difference in protein consumption between the sexes for Xiyasi individuals at any age). The average dentin δ^15^N after age 5 for Changxinyuan boys was 7.8‰, while the average girl's dentin δ^15^N after age 5 was 6.3‰. The nitrogen loess curve for Changxinyuan females is notably below the males, and this pattern suggests that Changxinyuan boys may have consumed more animal protein than girls and/or consumed proteins from higher trophic positions (with higher δ^15^N values). However, we caution that the Changxinyuan group is a small sample and therefore these findings may not be representative of the greater population dietary patterns.

#### 4.2.2 Carbon isotopes over the lifetime

During childhood, we see significant variability in δ^13^C dentin values, indicating urban Eastern Zhou children were fed across both C_3_ and C_4_ plant groups (Figure 3). Importantly, the δ^13^C values are generally more negative during childhood in comparison to the same individual's later adulthood bone collagen δ^13^C value. This indicates a greater consumption of C_3_ foods during childhood, and that as these individuals aged their diets changed and incorporated more C_4_ foods. We suggest that specific C_3_ foods such as wheat and soybeans were given to young children as important components of their diet while they were growing and developing, and that in later life their diets shifted to include more C_4_ foods (millets) which became registered in their bone collagen before death.

We also see that during childhood, the δ^13^C values of boys are slightly higher than the values of girls, indicating that boys are being fed more C_4_ foods than their sisters, cousins, and female peers. The average dentin δ^13^C value of Xiyasi boys (after age 5) was −13.6‰ while the average Xiyasi girl dentin δ^13^C after age 5 was −14.8‰, indicating greater consumption of C_4_ foods by Xiyasi boys than girls. We can see this pattern in the loess curve plotted in Figure 2, where the male blue curve is higher after weaning than the female curve, indicating more influence from C_4_ foods for boys. Within the Changxinyuan group, two of the males (CxyM049, CxyM026) show a different pattern, with dentin δ^13^C values that are mostly less negative during childhood than their adulthood bone collagen values, indicating greater C_4_ (millet) consumption during childhood followed by a slightly more mixed diet with more C_3_ inputs during adulthood. There is a very notable difference in the δ^13^C values between boys and girls for Changxinyuan: the average δ^13^C for girl's dentin (after age 5) is −14.2‰, while the average δ^13^C for boy's dentin (after age 5) is −9.8‰. The carbon loess curves (Figure 2) for these children show a significant divergence between the sexes. This large difference in carbon isotope values indicates significant dietary differences between females and males during childhood for these Changxinyuan individuals, with girls consuming much more C_3_ food than boys. We again must caveat that the Changxinyuan sample is small, but is suggestive of dramatic dietary differences between boys and girls beginning in early youth.

#### 4.2.3 Dietary differences between females and males over the lifetime

The patterning of δ^13^C dentin values during childhood relative to adulthood reveal that Eastern Zhou children's diets were distinct from adulthood diets, and that C_3_ foods were fed to children in greater quantities than what adults consumed (Figure 3). Taken together, these findings are significant because the previous isotopic study of these individuals noted significant dietary differences between males and females during adulthood (Dong et al., 2017), and the dentin data, therefore, suggest that some of these differences are rooted in feeding practices that begin in childhood. Wheat and soybeans may have specifically been used as weaning/childhood foods for Eastern Zhou peoples, with millets being consumed in greater quantities later in life. Comparing the childhood dentin isotope data to each individual's later adulthood bone isotope data show dietary changes occurred over the lifetime and also highlight increasing differentiation between males and females with age, suggesting that food practices were integral to aspects of gender over the lifetime (Appadurai, 1981; Hastorf, 2017; Miller et al., 2018; Richards, 1951; Twiss, 2007; Weismantel, 1988; White, 2005).

Beginning in childhood, boys were fed more millet which then increased significantly at a later point in their lives. One possibility is that dietary changes for males were tied to social age/status changes over their lifetime, such as the coming‐of‐age “capping” ritual for adolescent/young adult men (Chu & Buckley Ebrey, 1991; Hardy, 1993; Marshall, 2003; Rouzer, 2001). This ritual is written about more extensively in the Han Dynasty period but is believed to have had its roots in earlier periods, perhaps beginning in the Bronze Age Eastern Zhou (Hardy, 1993). Males would be ceremoniously capped (in their later teenage years) in a ritual marking their transition into adulthood and heralding their new responsibilities and rights that change in status afforded them (Chu & Buckley Ebrey, 1991; Hardy, 1993; Marshall, 2003; Rouzer, 2001). Perhaps dietary changes accompanied this social age transition to adulthood and Eastern Zhou young men then consumed greater amounts of millet as adults (potentially both as food and in fermented alcoholic beverages). This is a hypothesis that could be tested with a targeted sampling of adult males who died across different age groups using both bone and multiple tooth samples (such as later forming third molars which potentially could capture the period pre‐ and post‐capping).

In childhood, Eastern Zhou girls ate across C_3_ and C_4_ food groups but consumed more C_3_ foods such as wheat and soybean than boys, which continued for these girls into adulthood. One explanation for the retention of more C_3_ foods in the diets of women may be related to gender divisions of labor, with women responsible for domestic work including household maintenance, food preparation, child care responsibilities, and textile production (Feng, 2013; Hinsch, 2018; Linduff & Sun, 2004; von Falkenhausen, 2006). Archaeological evidence of a gendered division of labor has primarily been inferred from objects found in tombs (Hinsch, 2018; Linduff & Sun, 2004; von Falkenhausen, 2006), however recent bioarchaeological analyses have made significant contributions to our understandings of gendered labor as well (He & Tang, 2015; Linduff & Sun, 2004; Mu & Chen, 2018; Zhang et al., 2017). Additionally, historical Chinese texts suggest that during the Eastern Zhou, women and men's daily lives were increasingly structured along divisions of labor which kept the sexes separated in time and space (“men grow grain and women produce cloth” 男耕女织; Hinsch, 2003, p. 598), and this may have had gendered consequences on the daily dietary practices of each sex (Hinsch, 2018; Linduff & Sun, 2004). If women's roles primarily focused on activities typically relegated to the domestic sphere (Hinsch, 2003, 2018; Linduff & Sun, 2004), and women were the primary cooks for the family, they may have eaten the same meals they fed to their children. Perhaps their husband ate most of these same meals as well, but may have received slightly different portions of particular parts of the meals, such as slightly more meat, and/or more millet, which would cause small but important changes to their bodily isotope chemistry, differentiating Eastern Zhou boys and men from girls and women. Further, it is common for caretakers to demonstrate to young children that a particular meal is good by eating some of it themselves (parental/social modeling), and this may provide one explanation for the retention of more C_3_ foods in female diets over the lifetime (Birch, 1999; Birch, Savage, & Ventura, 2007; Blissett & Fogel, 2013; Larsen et al., 2015). Taken together, these dietary data indicate that practices of differentiation between boys and girls began early in life through dietary habits where particular foods were associated with gender, and these gendered dietary differences intensified over the lifetime leading to the significant dietary differences noted between urban Eastern Zhou adult men and women (Dong et al., 2017).

The Zhou periods are now viewed as a major cultural shift in early Chinese history, with significant changes to ideology, religion and ritual, and political governing (Feng, 2013; Hinsch, 2018; von Falkenhausen, 2006). Separation of the sexes became increasingly codified in early Chinese society, particularly during the Zhou periods in response to what had been seen as cultural indulgences (such as excessive alcohol consumption) of the earlier Shang Dynasty elites (Feng, 2013; Hinsch, 2003, 2018). Coming‐of‐age rituals, such as capping for men and pinning for women (a ceremony at age 15 involving a change in hairstyle and signifying a social age status change to adulthood for young women), served to reinforce social norms differentiating between the sexes and their roles within the family and the greater social order (Chu & Buckley Ebrey, 1991; Hardy, 1993; Hinsch, 2003, 2018). Hinsch (2003, 2018) explains that the separate spheres of women's and men's daily lives were seen as an important aspect of Chinese cultural identity from this time period (from Eastern Zhou and into the Han Dynasty) and therefore we may be seeing dietary effects of these societal divisions in the isotopic data recorded in the teeth and bones from Xiyasi and Changxinyuan individuals.

### 4.3 A possible case of early life stress

One male individual, Xiyasi M205, had an exceptionally high dentin nitrogen value which stands out from the rest of the group. The first dentin sample for this individual (estimated median age of sample = 4 months) has a δ^15^N value of 15.0‰, which is 3‰ higher than the next highest δ^15^N value in the samples analyzed in this study. The second sample for Xiyasi M205 drops to δ^15^N = 9.7‰, at estimated age 1.2 years, and then maintains a relatively steady lower δ^15^N value for the subsequent years sampled (average δ^15^N value after age 5 years = 8.5‰). The first tooth section analyzed was formed during early infancy and therefore the high nitrogen value is a product of something that occurred in the first months after birth. There are two main theories that are typically proposed to explain high nitrogen values in infants: (a) the mother had a protein‐rich diet, potentially consuming marine food sources (or consumed other high isotope value protein sources), or (b) the infant experienced an intense period of physiological stress (Beaumont & Montgomery, 2016; Halcrow et al., 2018; King et al., 2018; Mbeki, Kootker, Kars, & Davies, 2017; Mekota, Grupe, Ufer, & Cuntz, 2006; Neuberger, Jopp, Graw, Püschel, & Grupe, 2013). We believe that the second option, physiological stress, is likely the cause of Xiyasi M205's high nitrogen value from the first months of life. Although we cannot rule out that this boy's mother may have had a very unusual diet (she would have been an outlier relative to the rest of the population under study to have had δ^15^N in the range high enough to have a breastfeeding baby reach δ^15^N = 15‰) we think this is unlikely given that the boy's second dentin sample drops significantly and then continues to slowly decline and stabilize, suggesting that breast milk was still a portion of his diet and therefore his mother's nitrogen values were similar to the rest of the adult female population (around 7–8‰). We believe the high nitrogen isotope value reflects a brief but significant period of stress after birth where the infant was being breast‐fed but also was catabolizing his own bodily tissues to provide him sufficient energy requirements for survival (Beaumont & Montgomery, 2016; King et al., 2018; Mekota et al., 2006; Neuberger et al., 2013). Luckily, this period was short‐term and likely concluded before his first birthday as his second dentin sample shows a much lower nitrogen value, within the range of his peers. Xiyasi M205 survived this early‐life stress event and lived into older age, dying after the age of 50, and was given a moderate level of mortuary treatment. Future study of linear enamel hypoplasia and other pathological skeletal indicators may further illuminate the roles of diet and nutrition on physiological stress for these communities.

## 5 CONCLUSIONS

Feeding children goes beyond biological necessity and into the realms of sociopolitical action: what one feeds a developing body needs to be both nutritionally adequate and culturally acceptable (Bentley et al., 1991). Therefore, examining childhood dietary practices provides a unique perspective on a critical period of human development where a body is being fed both to grow healthily and to grow into a body that is socially appropriate. Using isotopic data, we can reconstruct breastfeeding, weaning, and childhood dietary practices for archaeological populations and gain important insights into how ancient peoples raised their families and helped them grow in socially meaningful ways. In this study we have seen how people living in the ancient Zhenghan city during the Eastern Zhou period had access to an array of foods but that some of these foods were consumed in greater proportions during certain periods of their life, and some foods were also variably consumed based on an individual's sex.

We hypothesized that weaning was completed between 2 and 4 years of age, and our dentin isotopic data indicate that most Eastern Zhou children from these urban sites were weaned between 2.5 and 4 years, following the pattern documented in other ancient Chinese populations (Xia et al., 2018; Yi et al., 2018). We also hypothesized that dietary differences between females and males would begin in childhood and would be recorded as isotopically distinct diets, and indeed the isotopic data suggest that boys consumed more millet than girls beginning in childhood. Finally, the comparison of dentin and bone collagen isotope data from the same individuals show that there was significant dietary change over the lifetime for all people, with childhood diet reflecting greater wheat and soybean consumption, and adulthood diet shifting to greater millet consumption.

Many archaeologists are interested in the timing and reasoning for why particular plants are adopted and incorporated into cultures where they are novel and not natively cultivated but over time may become central components of local diet (Boivin, Fuller, & Crowther, 2012; Jones et al., 2011; Liu et al., 2014; Liu & Jones, 2014). Various theories about crop adoption have been proposed ranging from ecological theories (such as drought tolerance, crop yield relative to land usage, human/land energy efficiency models) to more socially‐oriented theories (such as exotic foods as high‐status foods which over time become normalized into general population diet). It is most probable that the incorporation of particular foods that ultimately become dietary staples reflects both ecological and human sociopolitical–ideological factors. During the Bronze Age Eastern Zhou period on the Central Plain of China, wheat and soybean were finding their place within regional Chinese cultures and cuisines, and perhaps for the people living in and around the ancient Zhenghan City, these new plants began to find their place with feeding children.

## Supporting information


**Table S1** supporting information

**Figure S1** Box plots of the estimated weaning ages of Xiyasi and Changxinyuan individuals based on the patterning of the dentin stable isotope data. Data from each site and each sex (females in red, males in blue) are separated to show the trends across each group.

## Data Availability

All data for this research is presented within the manuscript and supplementary materials available online.
