# Supplementary material for: Raising girls and boys in early China: Stable isotope data reveal sex differences in weaning and childhood diets during the eastern Zhou era
Source: Am J Phys Anthropol. 2020 Mar 6;172(4):567–85. doi: 10.1002/ajpa.24033 (PMC7496748; doi:10.1002/ajpa.24033)
Supplement: Supplementary file 1 — Table S1 supporting information [file AJPA-172-567-s002.docx]

| **Site** | **Feature** | **Species** | **d^13^C ‰** | **d^15^N ‰** | **C%** | **N%** | **C:N** |
| --- | --- | --- | --- | --- | --- | --- | --- |
| Changxinyuan | T40G4 | pig | -8.7 | 7.3 | 39.7 | 13.6 | 3.4 |
| Changxinyuan | M64 | pig | -10.2 | 5.4 | 47.8 | 17.5 | 3.2 |
| Changxinyuan | T38J104 | pig | -13.1 | 5.1 | 27.3 | 9.8 | 3.3 |
| Changxinyuan | T38J104 | dog | -15.7 | 6.9 | 44.8 | 15.2 | 3.4 |
| Changxinyuan | T30G4 | dog | -14.7 | 4.9 | 27.8 | 10.0 | 3.3 |
| Changxinyuan | M67 | dog | -11.6 | 5.7 | 44.9 | 15.2 | 3.4 |
| Changxinyuan | T30G4 | bovine | -11.7 | 7.2 | 27.1 | 10.0 | 3.2 |
| Changxinyuan | T38J104 | bovine | -10.4 | 2.4 | 47.2 | 17.4 | 3.2 |
| Tianli | M47 | pig | -13.9 | 5.3 | 39.8 | 14.5 | 3.2 |
| Tianli | M49 | pig | -7.9 | 7.7 | 40.1 | 14.7 | 3.2 |
| Tianli | M46 | pig | -14.4 | 6.1 | 40.5 | 14.6 | 3.2 |
| Tianli | M8 | pig | -13.5 | 6.2 | 39.9 | 14.4 | 3.2 |
| Tianli | M56 | dog | -9.4 | 7.3 | 40.9 | 14.8 | 3.2 |
| Tianli | M142 | bovine | -6.8 | 7.6 | 41.4 | 15.1 | 3.2 |
| Tianli | M152 | bovine | -10.4 | 7.7 | 41.5 | 15.3 | 3.2 |
| Tianli | M128 | bovine | -11.2 | 7.1 | 41.8 | 15.2 | 3.2 |
| Tianli | M142 | sheep | -16.6 | 7.9 | 41.3 | 15.0 | 3.2 |
| Tianli | M128 | sheep | -14.0 | 10.7 | 41.7 | 15.2 | 3.2 |
| Tianli | M55 | sheep | -14.9 | 8.6 | 41.2 | 15.1 | 3.2 |
| Tianli | M165 | sheep | -18.6 | 9.9 | 41.6 | 15.1 | 3.2 |
| Tianli | M142 | sheep | -17.0 | 6.6 | 41.7 | 15.3 | 3.2 |
| Tianli | M152 | sheep | -13.1 | 10.2 | 41.1 | 15.1 | 3.2 |

| **Individual ID** | **Tooth** | **Increment** | **Average age represented by increment (years)** | **%C** | **%N** | **C:N** | **𝞭**13C **(VPDB)** | **𝞭15N (AIR)** | **Weaning Age Estimate X = greater certainty given isotope pattern ? = less certainty** |
| --- | --- | --- | --- | --- | --- | --- | --- | --- | --- |
| Xiyasi_M139 | C | 1 | 0.6 | 39.6 | 14.6 | 3.2 | -12.7 | 10.0 |  |
| Xi_M139 | C | 2 | 1.5 | 39.1 | 15.0 | 3.0 | -13.5 | 9.5 |  |
| Xi_M139 | C | 3 | 2.5 | 43.8 | 14.8 | 3.4 | -16.9 | 8.3 |  |
| Xi_M139 | C | 4 | 3.4 | 42.5 | 15.1 | 3.3 | -16.5 | 7.4 |  |
| Xi_M139 | C | 5 | 4.3 | 43.5 | 15.4 | 3.3 | **-16.1** | **7.0** | **X** |
| Xi_M139 | C | 6 | 5.3 | 43.4 | 15.3 | 3.3 | -16.6 | 7.3 |  |
| Xi_M139 | C | 7 | 6.2 | 44.5 | 15.7 | 3.3 | -16.6 | 7.3 |  |
| Xi_M139 | C | 8 | 7.1 | 44.0 | 15.7 | 3.3 | -16.4 | 7.3 |  |
| Xi_M139 | C | 9 | 8.0 | 44.1 | 15.6 | 3.3 | -15.6 | 7.1 |  |
| Xi_M139 | C | 10 | 9.0 | 43.6 | 15.5 | 3.3 | -16.1 | 6.6 |  |
| Xi_M139 | C | 11 | 9.9 | 40.3 | 15.3 | 3.1 | -14.6 | 7.2 |  |
| Xi_M139 | C | 12 -- 13 | 11.3 | 41.3 | 14.8 | 3.3 | -15.2 | 7.5 |  |
| Xi_M139 | C | 14 -- 15 | 13.2 | 43.1 | 14.8 | 3.4 | -17.4 | 7.7 |  |
| Xiyasi_M237 | C | 1 | 2.8 | 45.2 | 15.5 | 3.4 | -15.7 | 7.1 |  |
| Xi_M237 | C | 2 | 3.5 | 45.2 | 15.7 | 3.4 | **-16.6** | **7.2** | **?** |
| Xi_M237 | C | 3 | 4.3 | 43.2 | 15.3 | 3.3 | -15.4 | 7.7 |  |
| Xi_M237 | C | 4 | 5.0 | 44.8 | 15.8 | 3.3 | -17.2 | 7.1 |  |
| Xi_M237 | C | 5 | 5.7 | 41.9 | 15.7 | 3.1 | -15.3 | 6.8 |  |
| Xi_M237 | C | 6 | 6.4 | 44.2 | 15.5 | 3.3 | -16.7 | 6.5 |  |
| Xi_M237 | C | 7 | 7.2 | 41.6 | 15.3 | 3.2 | -16.3 | 5.4 |  |
| Xi_M237 | C | 8 | 7.9 | 41.2 | 14.9 | 3.2 | -15.3 | 5.7 |  |
| Xi_M237 | C | 9 | 8.6 | 43.2 | 15.7 | 3.2 | -14.2 | 6.4 |  |
| Xi_M237 | C | 10 | 9.4 | 42.9 | 15.3 | 3.3 | -11.9 | 6.9 |  |
| Xi_M237 | C | 11 | 10.1 | 41.7 | 15.5 | 3.1 | -12.3 | 7.0 |  |
| Xi_M237 | C | 12 | 10.8 | 41.5 | 15.4 | 3.1 | -13.9 | 6.3 |  |
| Xi_M237 | C | 13 -- 14 | 11.9 | 41.3 | 15.1 | 3.2 | -14.2 | 6.8 |  |
| Xi_M237 | C | 15 -- 16 | 13.4 | 41.3 | 15.4 | 3.1 | -14.9 | 6.7 |  |
| Xiyasi_M088 | M1 | 1 | 0.3 | 41.6 | 15.5 | 3.1 | -15.7 | 10.9 |  |
| Xi_M088 | M1 | 2 | 1.1 | 40.0 | 15.1 | 3.1 | -16.5 | 10.1 |  |
| Xi_M088 | M1 | 3 | 1.8 | No Yield | | | | |  |
| Xi_M088 | M1 | 4 | 2.6 | 39.0 | 14.9 | 3.1 | -17.6 | 7.6 |  |
| Xi_M088 | M1 | 5 | 3.3 | 37.8 | 14.5 | 3.0 | **-17.9** | **7.4** | **X** |
| Xi_M088 | M1 | 6 | 4.1 | 43.4 | 15.9 | 3.2 | -16.1 | 7.5 |  |
| Xi_M088 | M1 | 7 | 4.8 | 38.8 | 14.5 | 3.1 | -19.2 | 8.0 |  |
| Xi_M088 | M1 | 8 | 5.6 | 37.9 | 14.6 | 3.0 | -18.7 | 7.9 |  |
| Xi_M088 | M1 | 9 | 6.3 | 41.4 | 15.5 | 3.1 | -16.9 | 7.7 |  |
| Xi_M088 | M1 | 10 | 7.1 | 40.6 | 15.2 | 3.1 | -17.0 | 8.1 |  |
| Xi_M088 | M1 | 11 | 7.8 | 40.3 | 15.4 | 3.1 | -17.2 | 9.0 |  |
| Xi_M088 | M1 | 12 -- 13 | 9.0 | 39.9 | 15.1 | 3.1 | -18.6 | 9.2 |  |
| Xiyasi_M157 | C | 1 | 2.5 | 41.6 | 15.7 | 3.1 | -11.1 | 7.5 |  |
| Xi_M157 | C | 2 | 3.3 | 39.5 | 15.3 | 3.0 | -13.3 | 6.9 |  |
| Xi_M157 | C | 3 | 4.1 | 38.8 | 14.7 | 3.1 | **-12.8** | **6.7** | **X** |
| Xi_M157 | C | 4 | 4.9 | 40.5 | 15.5 | 3.1 | -11.8 | 6.8 |  |
| Xi_M157 | C | 5 | 5.7 | 39.6 | 14.9 | 3.1 | -11.8 | 6.9 |  |
| Xi_M157 | C | 6 | 6.6 | 41.6 | 15.8 | 3.1 | -14.4 | 6.7 |  |
| Xi_M157 | C | 7 | 7.4 | 41.0 | 15.7 | 3.0 | -14.4 | 6.1 |  |
| Xi_M157 | C | 8 | 8.2 | 39.4 | 15.3 | 3.0 | -15.7 | 5.6 |  |
| Xi_M157 | C | 9 | 9.0 | 40.5 | 15.8 | 3.0 | -15.6 | 5.5 |  |
| Xi_M157 | C | 10 | 9.8 | 39.7 | 15.4 | 3.0 | -14.4 | 5.3 |  |
| Xi_M157 | C | 11 | 10.6 | 39.8 | 15.6 | 3.0 | -12.9 | 5.7 |  |
| Xi_M157 | C | 12 -- 13 | 11.8 | 41.7 | 15.7 | 3.1 | -11.6 | 6.7 |  |
| Xiyasi_M350 | M1 | 1 | 1.1 | 40.1 | 15.4 | 3.0 | -9.3 | 11.1 |  |
| Xi_M350 | M1 | 2 | 1.8 | 41.2 | 15.8 | 3.0 | -11.9 | 8.3 |  |
| Xi_M350 | M1 | 3 | 2.6 | 39.5 | 15.4 | 3.0 | -12.8 | 8.3 |  |
| Xi_M350 | M1 | 4 | 3.4 | 39.6 | 15.4 | 3.0 | **-13.0** | **8.0** | **?** |
| Xi_M350 | M1 | 5 | 4.2 | 40.2 | 15.7 | 3.0 | -13.8 | 7.5 |  |
| Xi_M350 | M1 | 6 | 4.9 | 38.9 | 15.4 | 3.0 | -13.7 | 6.8 |  |
| Xi_M350 | M1 | 7 | 5.7 | 38.2 | 15.1 | 2.9 | -13.4 | 7.1 |  |
| Xi_M350 | M1 | 8 | 6.5 | 40.2 | 15.3 | 3.1 | -11.7 | 7.0 |  |
| Xi_M350 | M1 | 9 | 7.2 | No Yield | | | | |  |
| Xi_M350 | M1 | 10 -- 11 | 8.4 | 38.5 | 15.0 | 3.0 | -13.6 | 6.7 |  |
| Xiyasi_M051 | M1 | 1 | 0.3 | 40.5 | 15.7 | 3.0 | -10.8 | 11.1 |  |
| Xi_M051 | M1 | 2 | 1.0 | 40.5 | 15.3 | 3.1 | -11.8 | 10.3 |  |
| Xi_M051 | M1 | 3 | 1.6 | 40.0 | 15.6 | 3.0 | -15.0 | 8.8 |  |
| Xi_M051 | M1 | 4 | 2.3 | 39.4 | 15.5 | 3.0 | **-15.4** | **8.5** | **?** |
| Xi_M051 | M1 | 5 | 2.9 | 38.5 | 15.3 | 2.9 | -16.5 | 7.6 |  |
| Xi_M051 | M1 | 6 | 3.6 | 39.4 | 15.5 | 3.0 | -16.8 | 7.4 |  |
| Xi_M051 | M1 | 7 | 4.3 | 41.1 | 15.7 | 3.1 | -14.4 | 7.3 |  |
| Xi_M051 | M1 | 8 | 4.9 | 40.7 | 15.8 | 3.0 | -14.5 | 7.3 |  |
| Xi_M051 | M1 | 9 | 5.6 | No Yield | | | | |  |
| Xi_M051 | M1 | 10 | 6.2 | 39.1 | 15.5 | 2.9 | -17.2 | 6.9 |  |
| Xi_M051 | M1 | 11 -- 12 | 7.2 | 40.7 | 15.9 | 3.0 | -16.4 | 7.0 |  |
| Xi_M051 | M1 | 13 -- 14 | 8.6 | 40.7 | 16.0 | 3.0 | -16.2 | 7.5 |  |
| Xiyasi_M162 | M1 | 1 | 1.1 | 41.7 | 16.0 | 3.0 | -12.9 | 9.5 |  |
| Xi_M162 | M1 | 2 | 1.8 | 39.5 | 15.9 | 2.9 | -15.3 | 9.0 |  |
| Xi_M162 | M1 | 3 | 2.6 | 40.1 | 15.9 | 2.9 | -16.1 | 7.1 |  |
| Xi_M162 | M1 | 4 | 3.3 | 39.4 | 15.7 | 2.9 | **-14.7** | **6.9** | **X** |
| Xi_M162 | M1 | 5 | 4.1 | 40.0 | 15.7 | 3.0 | -13.0 | 7.4 |  |
| Xi_M162 | M1 | 6 | 4.8 | 39.6 | 15.9 | 2.9 | -14.0 | 7.1 |  |
| Xi_M162 | M1 | 7 | 5.6 | No Yield | | | | |  |
| Xi_M162 | M1 | 8 | 6.3 |  |  |  |  |  |  |
| Xi_M162 | M1 | 9 | 7.1 |  |  |  |  |  |  |
| Xi_M162 | M1 | 10 | 7.8 | 37.8 | 15.0 | 2.9 | -13.6 | 8.1 |  |
| Xi_M162 | M1 | 11 -- 12 | 9.0 | 39.7 | 15.7 | 2.9 | -15.0 | 7.8 |  |
| Xiyasi_M094 | M1 | 1 | 0.3 | 40.3 | 15.9 | 3.0 | -15.1 | 11.3 |  |
| Xi_M094 | M1 | 2 | 1.0 | 40.3 | 16.1 | 2.9 | -15.1 | 10.0 |  |
| Xi_M094 | M1 | 3 | 1.6 | 41.8 | 16.4 | 3.0 | -14.6 | 10.5 |  |
| Xi_M094 | M1 | 4 | 2.3 | 38.0 | 14.5 | 3.1 | -12.9 | 10.1 |  |
| Xi_M094 | M1 | 5 | 2.9 | 39.8 | 16.1 | 2.9 | -15.5 | 9.4 |  |
| Xi_M094 | M1 | 6 | 3.6 | 40.5 | 16.3 | 2.9 | -16.5 | 8.9 |  |
| Xi_M094 | M1 | 7 | 4.3 | 36.2 | 14.7 | 2.9 | **-16.3** | **8.8** | **X** |
| Xi_M094 | M1 | 8 | 4.9 | 40.4 | 16.4 | 2.9 | -16.2 | 9.0 |  |
| Xi_M094 | M1 | 9 | 5.6 | 39.6 | 15.8 | 2.9 | -15.3 | 8.7 |  |
| Xi_M094 | M1 | 10 | 6.2 | 37.8 | 15.1 | 2.9 | -14.6 | 8.7 |  |
| Xi_M094 | M1 | 11 | 6.9 | 40.3 | 15.8 | 3.0 | -11.8 | 9.3 |  |
| Xi_M094 | M1 | 12 | 7.6 | 39.4 | 15.8 | 2.9 | -11.8 | 9.2 |  |
| Xi_M094 | M1 | 13 -- 14 | 8.5 | 41.3 | 15.8 | 3.1 | -13.8 | 10.0 |  |
| Xiyasi_M016 | M1 | 1 | 0.3 | 42.8 | 15.8 | 3.2 | -10.5 | 10.6 |  |
| Xi_M016 | M1 | 2 | 1.2 | 40.9 | 15.4 | 3.1 | -10.5 | 9.7 |  |
| Xi_M016 | M1 | 3 | 2.1 | 40.9 | 15.6 | 3.0 | -11.1 | 9.5 |  |
| Xi_M016 | M1 | 4 | 2.9 | 41.1 | 15.5 | 3.1 | -11.3 | 7.9 |  |
| Xi_M016 | M1 | 5 | 3.8 | 42.4 | 15.6 | 3.2 | **-9.7** | **7.6** | **X** |
| Xi_M016 | M1 | 6 | 4.7 | 40.9 | 15.7 | 3.0 | -10.0 | 7.8 |  |
| Xi_M016 | M1 | 7 | 5.6 | No Yield | | | | |  |
| Xi_M016 | M1 | 8 | 6.5 | 40.6 | 15.5 | 3.1 | -10.2 | 8.2 |  |
| Xi_M016 | M1 | 9 | 7.3 | 40.3 | 15.9 | 3.0 | -12.2 | 7.8 |  |
| Xi_M016 | M1 | 10 | 8.2 | No Yield | | | | |  |
| Xi_M016 | M1 | 11 | 9.1 | 41.9 | 15.9 | 3.1 | -9.3 | 8.5 |  |
| Xiyasi_M018 | M1 | 1 | 0.3 | 42.6 | 16.1 | 3.1 | -11.5 | 11.1 |  |
| Xi_M018 | M1 | 2 | 1.2 | 43.6 | 15.9 | 3.2 | -9.5 | 10.7 |  |
| Xi_M018 | M1 | 3 | 2.1 | 41.1 | 15.8 | 3.0 | -12.2 | 10.4 |  |
| Xi_M018 | M1 | 4 | 3.1 | 42.0 | 16.0 | 3.1 | **-11.7** | **9.3** | **?** |
| Xi_M018 | M1 | 5 | 4.0 | No Yield | | | | |  |
| Xi_M018 | M1 | 6 | 4.9 |  |  |  |  |  |  |
| Xi_M018 | M1 | 7 | 5.8 | 42.6 | 15.9 | 3.1 | -9.2 | 8.6 |  |
| Xi_M018 | M1 | 8 | 6.7 | 40.8 | 15.7 | 3.0 | -11.1 | 8.5 |  |
| Xi_M018 | M1 | 9 | 7.7 | No Yield | | | | |  |
| Xi_M018 | M1 | 10 | 8.6 |  |  |  |  |  |  |
| Xiyasi_M040 | C | 1 | 2.6 | 42.6 | 15.8 | 3.1 | -14.2 | 7.9 |  |
| Xi_M040 | C | 2 | 3.6 | 41.4 | 15.8 | 3.1 | **-14.6** | **7.7** | **?** |
| Xi_M040 | C | 3 | 4.6 | 41.2 | 15.7 | 3.1 | -13.4 | 8.3 |  |
| Xi_M040 | C | 4 | 5.6 | 37.3 | 14.8 | 2.9 | -14.3 | 7.1 |  |
| Xi_M040 | C | 5 | 6.5 | 42.5 | 15.9 | 3.1 | -14.2 | 7.1 |  |
| Xi_M040 | C | 6 | 7.5 | 41.1 | 16.0 | 3.0 | -14.4 | 7.4 |  |
| Xi_M040 | C | 7 | 8.5 | 41.2 | 15.9 | 3.0 | -12.9 | 8.0 |  |
| Xi_M040 | C | 8 | 9.5 | 42.6 | 16.0 | 3.1 | -13.2 | 8.1 |  |
| Xi_M040 | C | 9 | 10.5 | 41.3 | 15.5 | 3.1 | -15.1 | 7.7 |  |
| Xi_M040 | C | 10 | 11.5 | 42.6 | 15.8 | 3.1 | -14.6 | 7.8 |  |
| Xi_M040 | C | 11 -- 12 | 13.0 | 39.7 | 15.2 | 3.1 | -15.5 | 7.0 |  |
| Xiyasi_M205 | M1 | 1 | 0.3 | 42.1 | 15.6 | 3.2 | -12.1 | 15.0 |  |
| Xi_M205 | M1 | 2 | 1.2 | 41.6 | 16.0 | 3.0 | -14.6 | 9.7 |  |
| Xi_M205 | M1 | 3 | 2.1 | 42.2 | 15.8 | 3.1 | -12.8 | 9.0 |  |
| Xi_M205 | M1 | 4 | 3.1 | 41.5 | 15.9 | 3.0 | -11.9 | 8.6 |  |
| Xi_M205 | M1 | 5 | 4.0 | 42.8 | 16.1 | 3.1 | **-9.4** | **8.4** | **?** |
| Xi_M205 | M1 | 6 | 4.9 | 41.0 | 16.0 | 3.0 | -12.2 | 8.2 |  |
| Xi_M205 | M1 | 7 | 5.8 | 40.4 | 15.7 | 3.0 | -12.6 | 8.3 |  |
| Xi_M205 | M1 | 8 | 6.7 | No Yield | | | | |  |
| Xi_M205 | M1 | 9 -- 10 | 8.1 | 42.2 | 15.9 | 3.1 | -12.1 | 8.7 |  |
| Xiyasi_M314 | C | 1 | 0.9 | No Yield | | | | |  |
| Xi_M314 | C | 2 | 1.8 | 41.7 | 15.9 | 3.1 | -12.9 | 8.9 |  |
| Xi_M314 | C | 3 | 2.6 | 42.7 | 16.0 | 3.1 | -12.2 | 8.1 |  |
| Xi_M314 | C | 4 | 3.5 | 42.5 | 15.9 | 3.1 | -12.7 | 7.9 |  |
| Xi_M314 | C | 5 | 4.3 | 42.3 | 15.9 | 3.1 | -14.2 | 7.8 |  |
| Xi_M314 | C | 6 | 5.2 | 40.3 | 15.7 | 3.0 | **-16.0** | **6.5** | **?** |
| Xi_M314 | C | 7 | 6.1 | 41.7 | 16.2 | 3.0 | -15.6 | 6.5 |  |
| Xi_M314 | C | 8 | 6.9 | 40.3 | 15.6 | 3.0 | -12.7 | 7.1 |  |
| Xi_M314 | C | 9 | 7.8 | 41.0 | 15.9 | 3.0 | -11.9 | 7.3 |  |
| Xi_M314 | C | 10 | 8.6 | 39.2 | 15.3 | 3.0 | -13.2 | 6.5 |  |
| Xi_M314 | C | 11 -- 12 | 9.9 | 40.8 | 16.0 | 3.0 | -14.3 | 5.9 |  |
| Xi_M314 | C | 13 -- 14 | 11.6 | 40.3 | 15.2 | 3.1 | -13.8 | 6.4 |  |
| Xiyasi_M068 | M1 | 1 | 0.3 | 43.9 | 15.6 | 3.3 | -16.2 | 10.7 |  |
| Xi_M068 | M1 | 2 | 1.2 | 36.3 | 13.9 | 3.0 | -14.3 | 10.9 |  |
| Xi_M068 | M1 | 3 | 2.1 | 41.9 | 15.9 | 3.1 | -14.8 | 10.1 |  |
| Xi_M068 | M1 | 4 | 2.9 | 42.6 | 16.3 | 3.1 | -14.4 | 9.5 |  |
| Xi_M068 | M1 | 5 | 3.8 | 39.3 | 15.4 | 3.0 | **-15.5** | **8.6** | **X** |
| Xi_M068 | M1 | 6 | 4.7 | 40.0 | 15.8 | 3.0 | -15.0 | 9.0 |  |
| Xi_M068 | M1 | 7 | 5.6 | No Yield | | | | |  |
| Xi_M068 | M1 | 8 | 6.5 |  |  |  |  |  |  |
| Xi_M068 | M1 | 9 | 7.3 | 41.0 | 15.8 | 3.0 | -13.9 | 9.4 |  |
| Xi_M068 | M1 | 10 | 8.2 | 40.6 | 15.4 | 3.1 | -14.2 | 9.0 |  |
| Xi_M068 | M1 | 11 | 9.1 | 40.8 | 15.8 | 3.0 | -14.6 | 8.8 |  |
| Xiyasi_M121 | C | 1 | 0.6 | 41.4 | 16.0 | 3.0 | -10.7 | 10.3 |  |
| Xi_M121 | C | 2 | 1.5 | No Yield | | | | |  |
| Xi_M121 | C | 3 | 2.5 | 41.5 | 16.2 | 3.0 | -13.8 | 8.4 |  |
| Xi_M121 | C | 4 | 3.4 | 40.8 | 15.5 | 3.1 | -12.4 | 8.5 |  |
| Xi_M121 | C | 5 | 4.3 | 39.8 | 15.4 | 3.0 | **-14.1** | **8.1** | **?** |
| Xi_M121 | C | 6 | 5.3 | 40.2 | 15.3 | 3.1 | -16.9 | 8.1 |  |
| Xi_M121 | C | 7 | 6.2 | 40.4 | 15.7 | 3.0 | -17.1 | 7.8 |  |
| Xi_M121 | C | 8 | 7.1 | 40.7 | 15.8 | 3.0 | -17.8 | 6.8 |  |
| Xi_M121 | C | 9 | 8.0 | 41.5 | 16.0 | 3.0 | -17.8 | 6.5 |  |
| Xi_M121 | C | 10 | 9.0 | 41.7 | 16.0 | 3.0 | -17.5 | 6.1 |  |
| Xi_M121 | C | 11 | 9.9 | 40.9 | 15.7 | 3.0 | -17.0 | 6.6 |  |
| Xi_M121 | C | 12 -- 13 | 11.3 | 39.0 | 15.2 | 3.0 | -18.0 | 6.7 |  |
| Xi_M121 | C | 14 -- 15 | 13.1 | 39.7 | 15.3 | 3.0 | -17.5 | 7.5 |  |
| Changxinyuan_M065 | C | 1 | 2.4 | 41.2 | 15.8 | 3.0 | -12.4 | 7.4 |  |
| Cxy_M065 | C | 2 | 3.2 | 41.3 | 15.5 | 3.1 | -13.0 | 6.9 |  |
| Cxy_M065 | C | 3 | 3.9 | 40.9 | 15.8 | 3.0 | **-13.3** | **6.8** | **?** |
| Cxy_M065 | C | 4 | 4.7 | No Yield | | | | |  |
| Cxy_M065 | C | 5 | 5.5 | 41.5 | 15.4 | 3.2 | -14.0 | 7.4 |  |
| Cxy_M065 | C | 6 | 6.2 | 43.2 | 16.1 | 3.1 | -13.1 | 6.8 |  |
| Cxy_M065 | C | 7 | 7.0 | 41.6 | 15.8 | 3.1 | -13.9 | 6.4 |  |
| Cxy_M065 | C | 8 | 7.7 | 42.7 | 16.2 | 3.1 | -11.6 | 6.9 |  |
| Cxy_M065 | C | 9 | 8.5 | 42.2 | 14.9 | 3.3 | -13.6 | 6.7 |  |
| Cxy_M065 | C | 10 | 9.3 | 41.3 | 15.2 | 3.2 | -13.7 | 6.9 |  |
| Cxy_M065 | C | 11 | 10.0 | 42.0 | 14.9 | 3.3 | -14.1 | 6.6 |  |
| Cxy_M065 | C | 12 -- 13.5 | 11.2 | No Yield | | | | |  |
| Cxy_M065 | C | 13.5 -- 15 | 12.2 | 42.6 | 15.5 | 3.2 | -15.1 | 5.4 |  |
| Changxinyuan_M045 | M1 | 1 | 0.3 | 42.4 | 15.4 | 3.2 | -10.5 | 11.4 |  |
| Cxy_M045 | M1 | 2 | 1.2 | 42.5 | 15.2 | 3.3 | -10.8 | 10.0 |  |
| Cxy_M045 | M1 | 3 | 2.1 | 43.1 | 15.2 | 3.3 | -10.5 | 10.2 |  |
| Cxy_M045 | M1 | 4 | 2.9 | 41.7 | 15.1 | 3.2 | **-13.5** | **8.9** | **?** |
| Cxy_M045 | M1 | 5 | 3.8 | 41.4 | 15.2 | 3.2 | -13.0 | 8.2 |  |
| Cxy_M045 | M1 | 6 | 4.7 | 42.0 | 15.2 | 3.2 | -12.6 | 7.9 |  |
| Cxy_M045 | M1 | 7 | 5.6 | 41.8 | 15.3 | 3.2 | -13.2 | 7.5 |  |
| Cxy_M045 | M1 | 8 | 6.5 | 41.8 | 15.3 | 3.2 | -12.9 | 7.3 |  |
| Cxy_M045 | M1 | 9 | 7.3 | 40.9 | 15.0 | 3.2 | -12.2 | 7.2 |  |
| Cxy_M045 | M1 | 10 | 8.2 | No Yield | | | | |  |
| Cxy_M045 | M1 | 11 | 9.1 |  |  |  |  |  |  |
| Changxinyuan_M026 | M1 | 1 | 1.2 | 42.2 | 15.0 | 3.3 | -9.9 | 10.2 |  |
| Cxy_M026 | M1 | 2 | 2.1 | 43.2 | 15.1 | 3.3 | -10.1 | 9.1 |  |
| Cxy_M026 | M1 | 3 | 2.9 | 40.5 | 14.8 | 3.2 | -11.6 | 8.1 |  |
| Cxy_M026 | M1 | 4 | 3.8 | 41.5 | 14.9 | 3.3 | **-11.7** | **7.2** | **X** |
| Cxy_M026 | M1 | 5 | 4.7 | 42.9 | 14.7 | 3.4 | -9.7 | 7.5 |  |
| Cxy_M026 | M1 | 6 | 5.6 | 43.2 | 15.4 | 3.3 | -9.1 | 7.4 |  |
| Cxy_M026 | M1 | 7 | 6.5 | 42.8 | 15.3 | 3.3 | -9.1 | 7.4 |  |
| Cxy_M026 | M1 | 8 | 7.3 | 42.9 | 15.6 | 3.2 | -9.5 | 7.1 |  |
| Cxy_M026 | M1 | 9 | 8.2 | 42.4 | 15.3 | 3.2 | -9.6 | 7.0 |  |
| Cxy_M026 | M1 | 10 | 9.1 | 42.3 | 15.4 | 3.2 | -9.9 | 6.9 |  |
| Changxinyuan_M049 | M1 | 1 | 1.8 | 43.3 | 15.4 | 3.3 | -9.4 | 12.0 |  |
| Cxy_M049 | M1 | 2 | 2.6 | 42.4 | 15.4 | 3.2 | -9.0 | 11.0 |  |
| Cxy_M049 | M1 | 3 | 3.4 | 44.4 | 15.5 | 3.3 | -6.7 | 10.2 |  |
| Cxy_M049 | M1 | 4 | 4.2 | 40.1 | 14.3 | 3.3 | **-7.7** | **9.8** | **?** |
| Cxy_M049 | M1 | 5 | 4.9 | 43.0 | 15.8 | 3.2 | -8.3 | 9.5 |  |
| Cxy_M049 | M1 | 6 | 5.7 | 42.1 | 15.4 | 3.2 | -8.7 | 9.4 |  |
| Cxy_M049 | M1 | 7 | 6.5 | 43.0 | 15.4 | 3.3 | -7.3 | 9.7 |  |
| Cxy_M049 | M1 | 8 | 7.2 | 42.1 | 15.3 | 3.2 | -7.9 | 9.1 |  |
| Cxy_M049 | M1 | 9 | 8.0 | 43.0 | 15.3 | 3.3 | -7.2 | 9.2 |  |
| Cxy_M049 | M1 | 10 | 8.8 | 42.8 | 15.6 | 3.2 | -8.9 | 8.4 |  |
| Changxinyuan_M074 | M1 | 1 | 1.1 | 42.5 | 15.2 | 3.3 | -11.7 | 8.6 |  |
| Cxy_M074 | M1 | 2 | 1.8 | 42.4 | 15.5 | 3.2 | -12.2 | 8.4 |  |
| Cxy_M074 | M1 | 3 | 2.6 | 42.8 | 15.6 | 3.2 | -14.0 | 7.5 |  |
| Cxy_M074 | M1 | 4 | 3.4 | 43.1 | 15.4 | 3.3 | **-13.3** | **6.9** | **?** |
| Cxy_M074 | M1 | 5 | 4.2 | 42.3 | 15.3 | 3.2 | -13.1 | 6.8 |  |
| Cxy_M074 | M1 | 6 | 4.9 | 42.3 | 15.4 | 3.2 | -12.6 | 6.7 |  |
| Cxy_M074 | M1 | 7 | 5.7 | 42.1 | 15.2 | 3.2 | -13.2 | 6.9 |  |
| Cxy_M074 | M1 | 8 | 6.5 | No Yield | | | | |  |
| Cxy_M074 | M1 | 9 | 7.2 | 43.2 | 15.3 | 3.3 | -10.8 | 7.4 |  |
| Cxy_M074 | M1 | 10 | 8.0 | 43.9 | 15.4 | 3.3 | -9.4 | 7.6 |  |
| Cxy_M074 | M1 | 11 | 8.8 | No Yield | | | | |  |
| Changxinyuan_M048 | M1 | 1 | 0.3 | 41.6 | 15.0 | 3.2 | -13.1 | 8.8 |  |
| Cxy_M048 | M1 | 2 | 1.2 | 43.5 | 15.2 | 3.3 | -12.6 | 7.8 |  |
| Cxy_M048 | M1 | 3 | 2.1 | 42.0 | 15.1 | 3.2 | **-14.7** | **6.6** | **?** |
| Cxy_M048 | M1 | 4 | 2.9 | 43.0 | 15.4 | 3.3 | -14.9 | 6.1 |  |
| Cxy_M048 | M1 | 5 | 3.8 | 42.3 | 15.3 | 3.2 | -15.6 | 5.7 |  |
| Cxy_M048 | M1 | 6 | 4.7 | 42.1 | 15.2 | 3.2 | -16.3 | 5.4 |  |
| Cxy_M048 | M1 | 7 | 5.6 | 41.2 | 14.7 | 3.3 | -15.9 | 5.5 |  |
| Cxy_M048 | M1 | 8 | 6.5 | 43.0 | 15.4 | 3.3 | -15.9 | 5.7 |  |
| Cxy_M048 | M1 | 9 | 7.3 | 42.9 | 15.3 | 3.3 | -15.3 | 5.7 |  |
| Cxy_M048 | M1 | 10 | 8.2 | 43.5 | 15.4 | 3.3 | -14.7 | 5.8 |  |
| Cxy_M048 | M1 | 11 | 9.1 | 43.1 | 15.4 | 3.3 | -15.8 | 5.5 |  |
| Changxinyuan_M043 | C | 1 | 2.8 | 45.4 | 15.6 | 3.4 | -12.2 | 9.2 |  |
| Cxy_M043 | C | 2 | 3.7 | 43.3 | 15.4 | 3.3 | -11.6 | 9.1 |  |
| Cxy_M043 | C | 3 | 4.6 | 42.9 | 15.4 | 3.3 | **-13.3** | **7.1** | **?** |
| Cxy_M043 | C | 4 | 5.6 | 43.8 | 15.6 | 3.3 | -13.3 | 6.7 |  |
| Cxy_M043 | C | 5 | 6.5 | 39.7 | 14.5 | 3.2 | -15.0 | 6.3 |  |
| Cxy_M043 | C | 6 | 7.4 | 42.7 | 15.7 | 3.2 | -15.4 | 5.6 |  |
| Cxy_M043 | C | 7 | 8.3 | 42.4 | 15.0 | 3.3 | -14.4 | 5.2 |  |
| Cxy_M043 | C | 8 | 9.3 | 43.0 | 15.3 | 3.3 | -15.2 | 5.1 |  |
| Cxy_M043 | C | 9 | 10.2 | 43.0 | 15.3 | 3.3 | -14.9 | 5.0 |  |
| Cxy_M043 | C | 10 | 11.1 | 43.2 | 15.7 | 3.2 | -15.4 | 5.1 |  |
| Cxy_M043 | C | 11 | 12.1 | 42.0 | 15.2 | 3.2 | -14.7 | 5.4 |  |
| Changxinyuan_M067 | C | 1 | 0.9 | 42.1 | 15.4 | 3.2 | -9.3 | 11.7 |  |
| Cxy_M067 | C | 2 | 1.8 | 42.0 | 15.4 | 3.2 | -11.6 | 10.5 |  |
| Cxy_M067 | C | 3 | 2.6 | 38.7 | 13.9 | 3.2 | **-13.2** | **8.6** | **X** |
| Cxy_M067 | C | 4 | 3.5 | 36.9 | 13.5 | 3.2 | -13.1 | 8.7 |  |
| Cxy_M067 | C | 5 | 4.3 | 41.0 | 14.5 | 3.3 | -10.8 | 8.5 |  |
| Cxy_M067 | C | 6 | 5.2 | 41.1 | 14.2 | 3.4 | -10.5 | 8.0 |  |
| Cxy_M067 | C | 7 | 6.1 | 35.4 | 12.8 | 3.2 | -9.6 | 8.6 |  |
| Cxy_M067 | C | 8 | 6.9 | 38.5 | 13.9 | 3.2 | -12.0 | 7.2 |  |
| Cxy_M067 | C | 9 | 7.8 | 40.3 | 14.0 | 3.4 | -10.9 | 6.1 |  |
| Cxy_M067 | C | 10 | 8.6 | 41.1 | 14.3 | 3.3 | -10.1 | 6.8 |  |
| Cxy_M067 | C | 11 -- 12 | 9.9 | 38.4 | 13.7 | 3.3 | -10.0 | 7.3 |  |
| Cxy_M067 | C | 13 -- 14 | 11.7 | No Yield | | | | |  |
