# Supplementary figures and images for: Raising girls and boys in early China: Stable isotope data reveal sex differences in weaning and childhood diets during the eastern Zhou era
Source: Am J Phys Anthropol. 2020 Mar 6;172(4):567–85. doi: 10.1002/ajpa.24033 (PMC7496748; doi:10.1002/ajpa.24033)

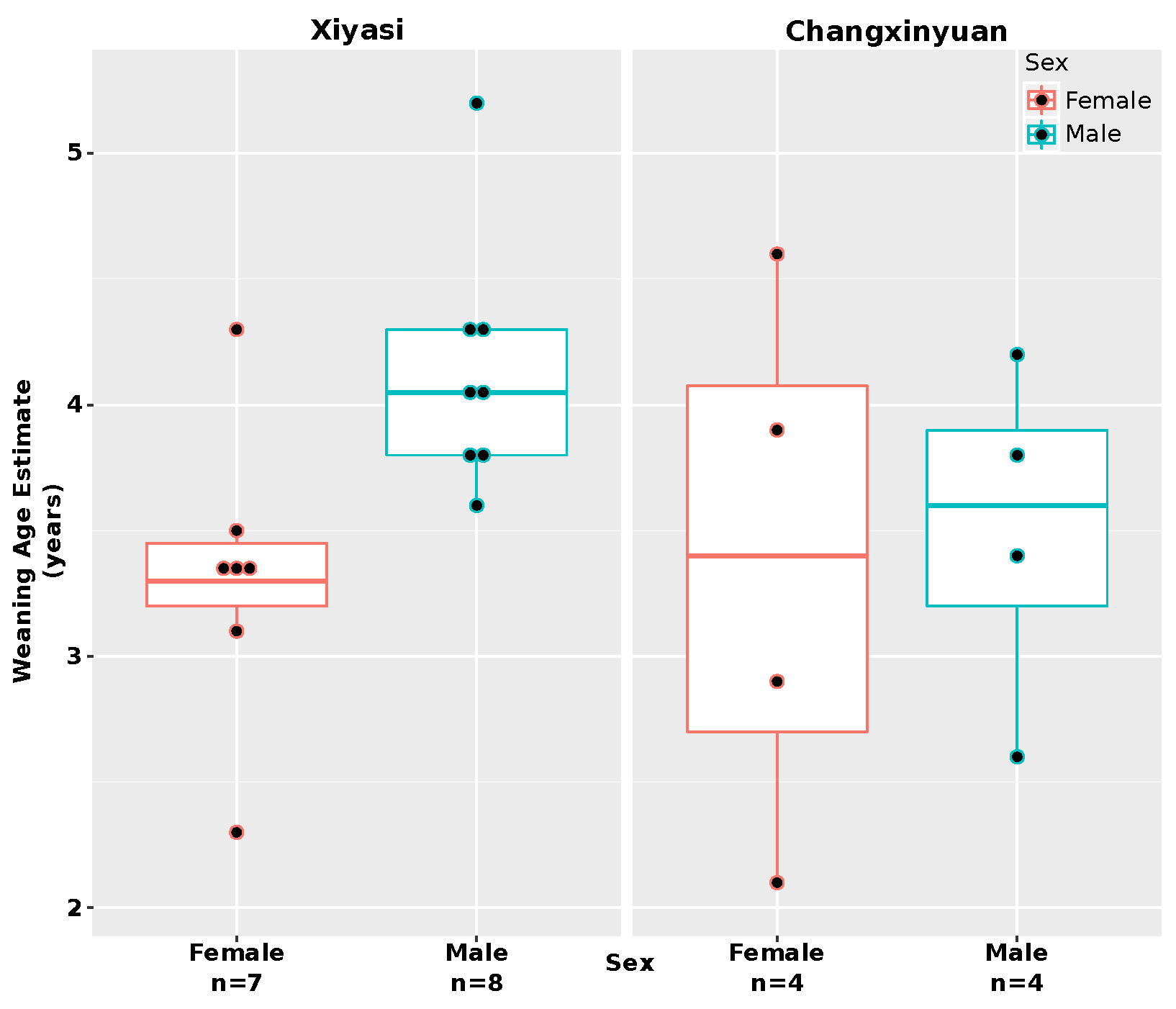

Supplement: Supplementary file 2 — Figure S1 Box plots of the estimated weaning ages of Xiyasi and Changxinyuan individuals based on the patterning of the dentin stable isotope data. Data from each site and each sex (females in red, males in blue) are separated to show the trends across each group. [file AJPA-172-567-s001.tif]
